# Iron‐Catalyzed Laser‐Induced Graphitization – Multiscale Analysis of the Structural Evolution and Underlying Mechanism

**DOI:** 10.1002/smll.202405558

**Published:** 2024-09-16

**Authors:** Christopher H. Dreimol, Ronny Kürsteiner, Maximilian Ritter, Annapaola Parrilli, Jesper Edberg, Jonas Garemark, Sandro Stucki, Wenqing Yan, Susanna Tinello, Guido Panzarasa, Ingo Burgert

**Affiliations:** ^1^ Wood Materials Science Institute for Building Materials ETH Zürich Zürich 8093 Switzerland; ^2^ Cellulose & Wood Materials Laboratory Empa – Swiss Federal Laboratories for Materials Science and Technology Dübendorf 8600 Switzerland; ^3^ Center for X‐ray Analytics Dübendorf 8600 Switzerland; ^4^ RISE Research Institutes of Sweden Digital Systems Smart Hardware Bio‐ and Organic Electronics Södra Grytsgatan 4 Norrköping 60233 Sweden; ^5^ Laboratory for Multifunctional Materials Department of Materials ETH Zürich Zürich 8093 Switzerland

**Keywords:** austenite, iron‐carbon composite, multilayer electrode, ostwald ripening, particle coalescence, sustainable electrode

## Abstract

The transition to sustainable materials and eco‐efficient processes in commercial electronics is a driving force in developing green electronics. Iron‐catalyzed laser‐induced graphitization (IC‐LIG) has been demonstrated as a promising approach for rendering biomaterials electrically conductive. To optimize the IC‐LIG process and fully exploit its potential for future green electronics, it is crucial to gain deeper insights into its catalyzation mechanism and structural evolution. However, this is challenging due to the rapid nature of the laser‐induced graphitization process. Therefore, multiscale preparation techniques, including ultramicrotomy of the cross‐sectional transition zone from precursor to fully graphitized IC‐LIG electrode, are employed to virtually freeze the IC‐LIG process in time. Complementary characterization is performed to generate a 3D model that integrates nanoscale findings within a mesoscopic framework. This enabled tracing the growth and migration behavior of catalytic iron nanoparticles and their role during the catalytic laser‐graphitization process. A three‐layered arrangement of the IC‐LIG electrode is identified including a highly graphitized top layer with an interplanar spacing of 0.343 nm. The middle layer contained γ‐iron nanoparticles encapsulated in graphitic shells. A comparison with catalyst‐free laser graphitization approaches highlights the unique opportunities that IC‐LIG offers and discuss potential applications in energy storage devices, catalysts, sensors, and beyond.

## Graphical abstract

Using multiscale characterization techniques, the growth and migration behavior of catalytic iron nanoparticles and elucidate are successfully traced to their role during Iron‐Catalyzed Laser‐Induced Graphitization (IC‐LIG), a promising approach for rendering biomaterials electrically conductive. Integrating nanoscale findings within a mesoscopic framework uncovers the structural evolution during graphitization and reveals the potential of this approach for future green electronics.

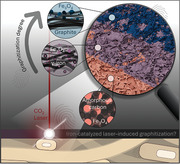

## 1 Introduction

The paradigm shift toward sustainable materials and eco‐efficient processes in electronics has been a driving force for innovative solutions in the development of green electronics. In this context, Laser‐Induced Graphitization (LIG) has emerged as a transformative processing technology that demonstrates the potential of using organic materials (e.g., wood,^[^ ^1^, ^2^, ^3^, ^4^, ^5^ ^]^ cellulose,^[^ ^6^, ^7^ ^]^ lignin,^[^ ^8^ ^]^ cork,^[^ ^9^ ^]^ chitin)^[^ ^10^ ^]^ as renewable precursors for highly conductive carbon‐based electrodes to replace the fossil‐based graphite most commonly used in commercial electronic products. This has resulted in the development of electrodes for biomedical applications,^[^ ^11^ ^]^ environmental (e.g., humidity)^[^ ^5^, ^12^ ^]^ and wearable sensors,^[^ ^7^, ^13^ ^]^ energy harvesting (e.g., triboelectric nanogenerators),^[^ ^14^ ^]^ and highly porous electrodes for energy storage devices (e.g., supercapacitors).^[^ ^7^, ^15^, ^16^ ^]^ Additional hybridization of wood‐based LIG electrodes with embedded metal nanocrystals (Cu, Co, Ni, Fe, NiFe) has been used for electrocatalytic applications in oxygen evolution reactions.^[^ ^2^ ^]^

**Figure 1:**
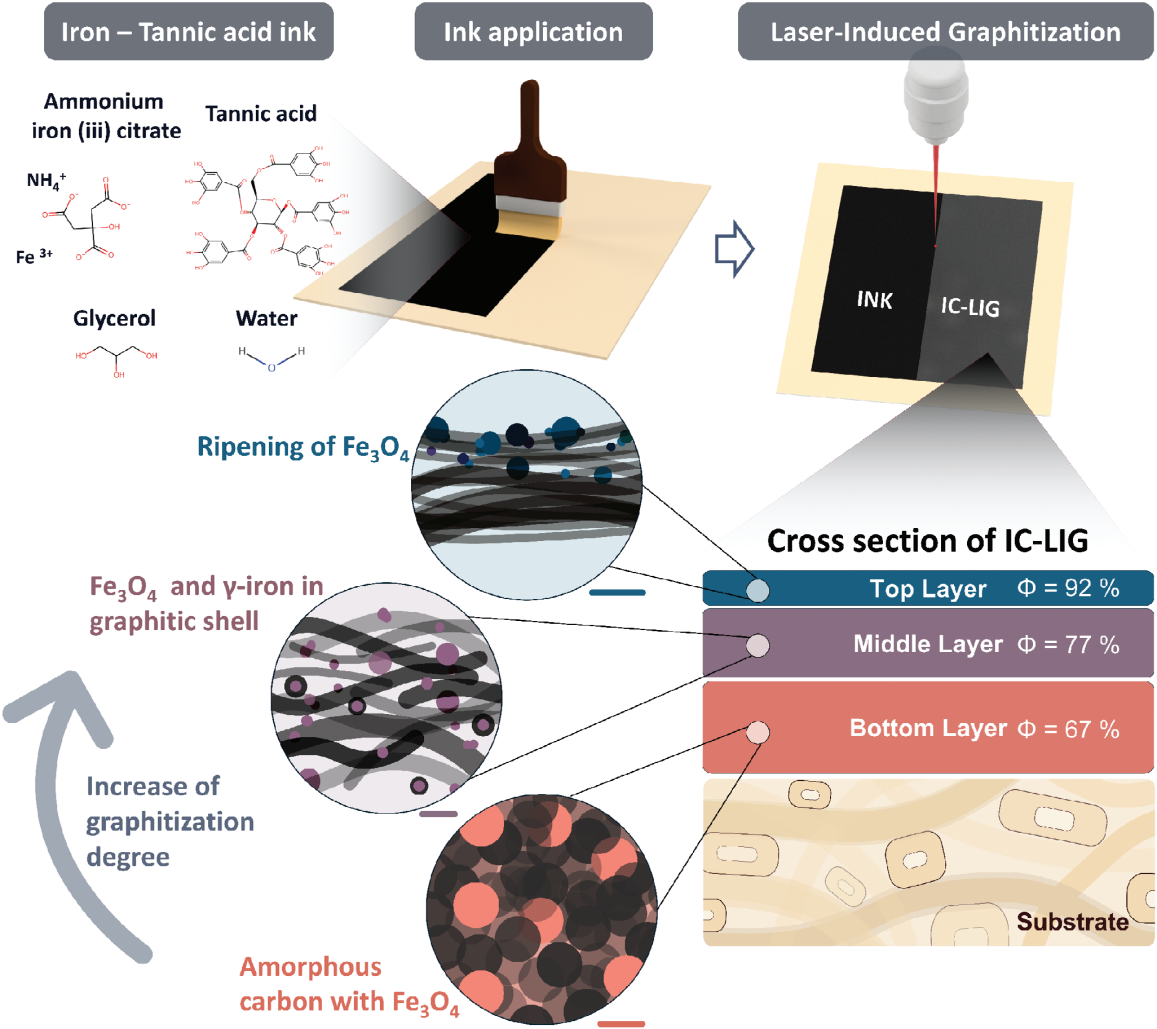
A schematic overview of the IC‐LIG process with the iron‐tannic acid ink and its ingredients, the ink application, and the laser graphitization process. The magnification schematically represents the IC‐LIG cross‐section with the observed layer arrangement across its thickness, highlighting individual structural features and the analyzed iron phase (insets). An increase in porosity (Φ) and graphitization degree was observed from the bottom to the top layer. The scale bars are 10 nm for the bottom layer, 50 nm for the middle layer, and 100 nm for the top layer.

Despite the great potential of laser‐induced graphitization technologies, current LIG processing routes have limited sustainability and scalability due to the use of flame retardants and solvents, lasing under an inert atmosphere (nitrogen and argon gas), and thermal (pre‐) processing steps for the pre‐carbonization of precursors. To address these challenges, we have recently introduced a novel approach called iron‐catalyzed laser‐induced graphitization (IC‐LIG, **Figure** **1**) and demonstrated its applicability by making wood surfaces electrically conductive.^[^ ^4^ ^]^ Coating a substrate with an aqueous iron‐containing bio‐based ink, followed by a CO_2_ laser treatment, allowed us to graphitize the surface of thermally sensitive substrates like wood and paper. Iron(III) ions and tannic acid are the essential components of the ink. Their combination results in a metal‐phenolic coordination complex that allows for catalytic graphitization of the ink‐coated substrate with a single laser step under air. Additionally, the ink compensates for any structural irregularities of the substrates used. The process was used to produce large (100 cm^2^), highly conductive (≥2500 S m^−1^; sheet resistance ≈18 Ω ◻^−1^) and homogeneous IC‐LIG electrodes.^[^ ^4^ ^]^

To optimize the IC‐LIG process and exploit its potential for green electronics applications, it is crucial to gain a deeper understanding of the mechanisms behind the IC‐LIG process. The scope of the present work is to gain fundamental insight into the catalyzation mechanism and the structural evolution of the IC‐LIG electrodes. We were especially interested in gaining insight into the role of iron as a catalyst during laser‐induced graphitization.

Therefore, we conducted a multiscale analysis (Figure S1, Supporting Information), with a particular emphasis on the structural evolution across the layers' thicknesses. Complementary characterization methods (Figure S1, Supporting Information), such as nano‐CT imaging techniques, allowed us to obtain a 3D reconstruction of the IC‐LIG electrode. This enabled us to incorporate the nanoscale findings and place them in a mesoscopic perspective as well as to trace the growth and migration behavior of the iron catalyst nanoparticles. Figure 1 provides a schematic overview of the identified layered arrangement of an IC‐LIG electrode across its thickness, highlighting individual structural features. In addition, we compare our laser‐induced graphitization process with conventional thermal transition metal catalysis‐based processes and catalyst‐free laser graphitization approaches, discussing the fundamental differences and mechanistic similarities between these methods and highlighting the unique opportunities presented by the iron‐catalyzed laser‐induced graphitization

## 2 Results and Discussion

The rough surface microstructure of natural materials, such as wood, can vary significantly between species and is influenced by the density (from the distribution of early‐ and late‐wood) and porosity (microstructural features such as the distribution of vessels (pores) in hardwoods).^[^ ^17^ ^]^ These variable features affect the thermal conductivity,^[^ ^18^ ^]^ leading to differences in heat dissipation during laser treatment. Therefore, to study the underlying mechanisms of the graphitization process, we selected a wood pulp board as a model substrate with a similar chemical composition but without the structural inhomogeneity of the wood surface. After applying the ink to the substrate, we graphitized the samples’ surface with a CO_2_ laser, according to our previously described procedure.^[^ ^4^ ^]^

During the IC‐LIG process, a highly porous carbonaceous material, here referred to as IC‐LIG electrode, is formed on the substrate surface (Figure 1). A key challenge of this investigation was to keep the entire IC‐LIG electrode unaffected and attached to the substrate. To preserve the IC‐LIG electrode during sample preparation, the IC‐LIG electrode together with the underlying substrate was embedded in a low‐viscosity resin that allowed for structure‐retaining ultrathin cross‐section cuts (≈100 nm) with an ultra‐microtome (Figure S2, Supporting Information), similar as previously described.^[^ ^19^ ^]^ This resulted in thin‐cut samples of the entire cross‐section incorporating the fully graphitized IC‐LIG electrode on its substrate for transmission electron microscopy (TEM) imaging. Additionally, block face samples of the transition zone from the bare precursor ink to the resulting fully graphitized IC‐LIG electrode were prepared. This allowed us to virtually freeze the IC‐LIG process in time for the subsequent investigation of the structural evolution and iron‐catalyzed laser‐induced graphitization mechanism.

**Figure 2:**
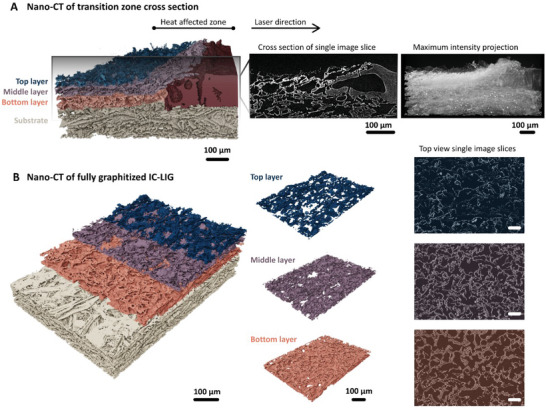
3D reconstruction of single nano‐CT images of the transition zone of cross‐sectional samples with corresponding single image slice and maximum intensity projection A). The 3D reconstruction highlights the transition from native ink to the development of a fully graphitized IC‐LIG electrode with its layers. Fully graphitized IC‐LIG electrode with separated layers and the corresponding nano‐CT image slice as a top view can be seen in B) highlighting the changes in porosity and varying intensities due to the different material composition allowing the separation of layers. The scale bar for nano‐CT top view single image slices is 100 µm.

A schematic overview of the sample preparation for the different analytical techniques used is shown in Figure S1 (Supporting Information), highlighting the strategy to cover different length scales from the meso‐ to the nanoscale. Starting at the mesoscale, bulk samples were laser‐cut from the transition zone and the fully graphitized area for subsequent nano‐CT analysis. The 3D image reconstructions obtained from the nano‐CT image acquisition revealed the structural evolution during the IC‐LIG electrode at the mesostructural level. **Figure** **2**A shows the transition zone of the laser‐induced graphitization process, starting with the thermal decomposition of the iron‐tannic acid ink and ending with the fully graphitized IC‐LIG electrode (from right to left). Here, the iron‐tannic acid ink precursor (Figure 2A; ruby‐colored layer) initially forms a homogeneous coating, while SEM images from the cross‐section revealed that ink‐encapsulated wood fibers are embedded in the iron‐tannic acid ink layer as well (Figure S5, Supporting Information blue squares).

**Figure 3:**
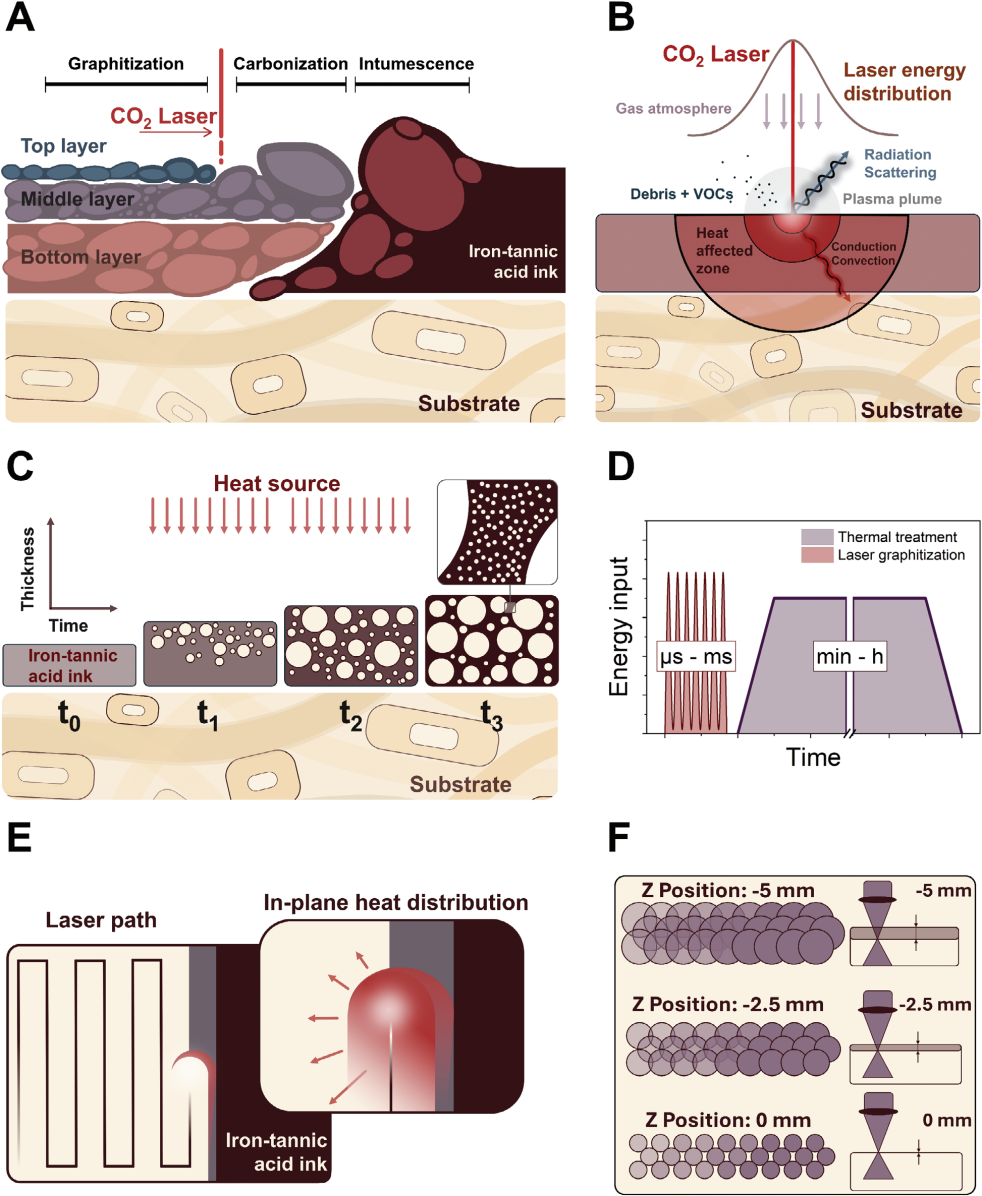
The illustration of the transition zone A) (cross‐sectional view) highlights the three different processing zones: the intumescence zone, carbonization zone, and graphitization zone, as well as the evolution of the layer arrangement as a function of laser position. In B), the laser beam‐material interactions are summarized, showing the Gaussian laser energy distribution and the resulting heat‐affected zone within the lasered material. The intumescent behavior of the iron‐tannic acid ink with bubble formation under a unidirectional heat source (C). In D) a comparison between conventional thermal treatments and laser graphitization highlights the differences in terms of their energy input over time. E) illustrates the laser path (engraving mode) and the in‐plane heat distribution as a result of the graphitization degree. F) highlights the dependence of focal depth on the focal area and overlaps of the resulting laser beam diameter.

Structural changes are already visible in the heat‐affected zone (Figure 2A) as the ink begins to form large pores, indicating strong intumescent behavior. We associate the formation of pores with the generation of volatile gases and the onset of melting and decomposition processes of the iron‐tannic acid ink. Nam et al. reported that the decomposition of tannic acid occurs at temperatures as low as 190 °C, and is associated with the formation of large pores ranging from 20 to 300 µm.^[^ ^20^ ^]^ Upon closer examination of the nano‐CT images of fully graphitized samples, a significant variation in pore size distribution was observed. Here, the microstructure can be described as a network of large cells together with smaller voids within the carbonaceous material. Structural changes can be further observed across the thickness of the sample. The upper surface of the carbonaceous material is rough (destruction of pore walls) and highly porous. As the structure progresses toward the substrate, the porous network becomes increasingly smooth and dense (Figure 2B; Figure S3 and Movie S1, Supporting Information). At the IC‐LIG‐substrate interface, individual wood fibers undergo a structural transformation, as their cell walls exhibit intumescent behavior as they are transformed into the IC‐LIG electrode, as evidenced by a sequence of top‐view nano‐CT images (Figure S4, Supporting Information, white box with dotted lines). Thus, they are involved in determining the overall porous structure and further facilitate the transition from wood pulp board to IC‐LIG electrode. It further indicates that the substrate serves not only structural functions but also acts as a direct precursor for IC‐LIG electrodes. We attribute the heterogeneous structure and structural changes across the sample thickness to a combination of intumescent behavior together with the unidirectional energy input from the CO_2_ laser (**Figure** **3**C), resulting in inhomogeneous heat dissipation during laser graphitization. Raman measurements from our previous study indicated a gradual decrease in the degree of graphitization moving from the top layer surface toward the substrate.^[^ ^4^ ^]^ Here, the top layer, which is the most exposed to the laser, was highly graphitized.

**Figure 4:**
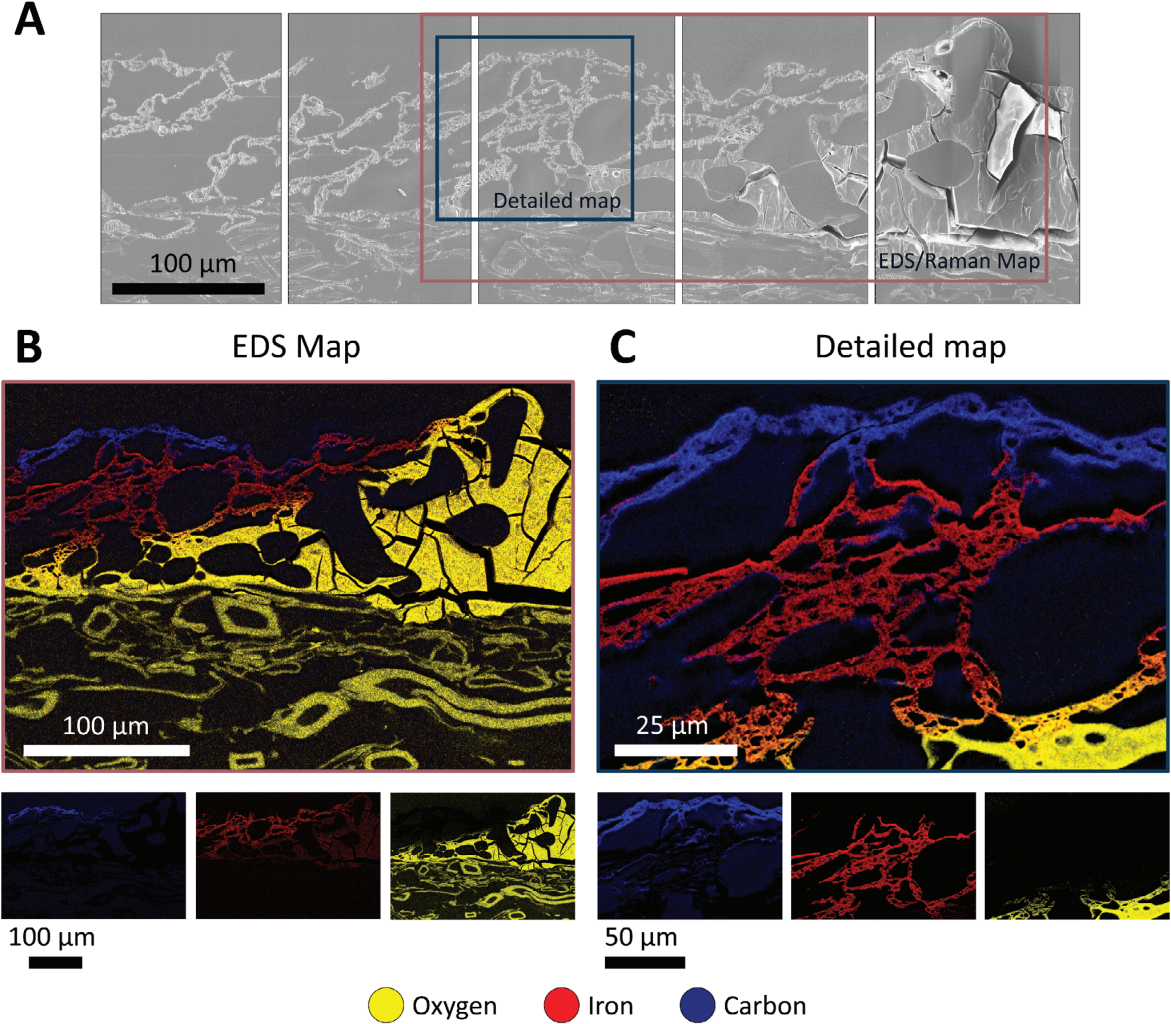
Panoramic view of scanning electron microscope images of the transition zone from a cross‐sectional view derived from block face samples A). An overview EDS map B) of the graphitized area and its corresponding detailed map C) (top and middle layer) highlight the distribution of oxygen (yellow), iron (red), and carbon (blue) (B). The same area of the detailed map was used for the AFM height map (Figure S13, Supporting Information).

Interestingly, we could also observe localized bright spots of higher intensity within the nano‐CT images (Figure 2B). Since iron species show high X‐ray attenuation, we assume that the observed bright spots are iron phases. The structural evolution with a layered arrangement, showing a high carbon content within the top layer and iron‐rich layers underneath, was further confirmed by scanning electron microscope (SEM) micrographs (**Figure** **4**A) together with the associated energy‐dispersive X‐ray spectroscopy (EDS) analysis (Figure 4B,C) and Raman map (Figures S6 and S7, Supporting Information) of the same block face samples. Interestingly, the changes in chemical composition are mainly recognizable in the reduction of the oxygen content together with the increase of the iron content from the iron‐tannic acid precursor ink to the fully graphitized IC‐LIG electrode. Such chemical transformation can be linked to the development of the intumescent layer during pyrolysis^[^ ^20^ ^]^ as well as the carbothermal reduction of iron oxides during the catalytic reactions.

We took advantage of the high X‐ray attenuation of iron species to investigate the layered arrangement in more detail. Figure 2A shows the 3D reconstruction of the transition zone derived from single nano‐CT image slices together with the maximum intensity projection (MIP, 2D projection as image overlay) obtained by projecting the brightest data value along each ray of sight. The intensity changes within the MIP image allowed for distinguishing between three layers. The varying intensities within these layers were attributed to different material compositions in the 3D model obtained from the nano‐CT analysis, allowing for their separation (Figure 2B; Figures S3 and S5, Supporting Information). Thus, we estimated the porosity of each layer and observed a decrease from the top to the middle and bottom layers to 92, 77, and 67%, respectively (Figure 1). The top views of each layer (Figure 2B; Figure S3 and S4, Supporting Information) show the changes in porosity, which originate from a combination of intumescent behavior, unidirectional energy input derived from the laser, and ablation during laser graphitization. Thereby, the top layer, which is most exposed to the high energy of the laser and a reactive oxygen‐rich atmosphere, experiences the greatest ablation.

It can be inferred that the unidirectional energy input from the laser‐induced graphitization, combined with the intumescent behavior of the iron‐tannic acid ink, has a substantial impact on structural evolution. We have therefore schematically abstracted the main characteristics in Figure 3 and related them to the microstructural changes during graphitization.

The transition from the native iron‐tannic acid ink to the fully graphitized IC‐LIG electrode, as a function of the heat generated by the laser, across three distinct processing zones is illustrated in Figure 3A. We define the first zone as the “intumescence zone” due to the formation of large pores, intumescence, and the onset of degradation of the iron‐tannic acid ink. It should be emphasized that the formation of pores, together with the intumescent behavior, already forms the resulting macrostructure within the intumescence zone. The structure will subsequently undergo laser‐induced carbonization and graphitization. From the EDS maps (**Figure** **4**B,C; Figure S8, Supporting Information), a porous char layer develops, as there is a sudden increase in carbon content together with a reduction of the oxygen content. This increase is associated with the degradation of the ink due to the onset of carbonization and is therefore defined as the beginning of the subsequent process zone, which we define as the “carbonization zone”. Once the laser has passed over the carbonization zone, the material experiences the greatest energy input due to the Gaussian energy distribution (Figure 3B). Consequently, the carbon content continues to increase, as evidenced by the significant increase in intensity in the EDS map (Figure 4B,C blue carbon layer). We therefore refer to this zone as the “graphitization zone.”

There are several parameters that affect the energy induced by a CO_2_ laser,^[^ ^21^ ^]^ the most important of which are power, velocity, and focal distance. For example, the influence of the focal distance on the energy distribution, as illustrated in Figure 3F. While focusing on the sample surface (z‐position: 0 mm) results in hardly any overlapping of the laser spots, changing the sample distance to not coincide with the focal distance (z‐position: −5 mm) can be used to induce an overlapping, thus reducing the energy induced on a single spot and allows for multiple lasing passes within a single laser cycle.^[^ ^22^ ^]^ The distribution of the heat to a greater area also has a direct influence on the graphitization, since it allows for a pre‐carbonization already in the heat‐affected zone^[^ ^22^, ^23^ ^]^ as also observed by the carbonization zone. Pre‐carbonization has an additional effect on heat transport across the thickness of the sample, as amorphous carbon acts as a thermal insulator,^[^ ^24^ ^]^ preventing the transfer of excessive heat into lower areas.

Similar to the defocus approach, multiple low‐energy irradiations can lead to a pre‐carbonization and subsequent LIG formation, while strong single irradiation can lead to pronounced chemical changes, degradation processes, and ablation. Here, the interplay of the applied energy with irradiation repetitions, for example, frequency (Hz) dithering (PPI, engraving mode), and velocity, becomes an important measure to tune the areal heat accumulation and graphitization process.^[^ ^25^ ^]^ Figure 3B illustrates the heat distribution across the sample thickness, which is represented by semicircular heat zones (heat‐affected zones). Thus, the strong demarcation of the three layers found in the nano‐CT image acquisition can be attributed to the different heat zones resulting from the unidirectional laser treatment (Figure 3B,C; Figure S5, Supporting Information). To provide a complementary investigation of the influence of the different heat zones across the sample thickness on the structural evolution, we prepared ultra‐thin sections for (S)TEM analysis and focused specifically on the phase as well as on the growth and migration behavior of the iron catalyst nanoparticles in the individual layers.

### 2.1 Top Layer

**Figure 5:**
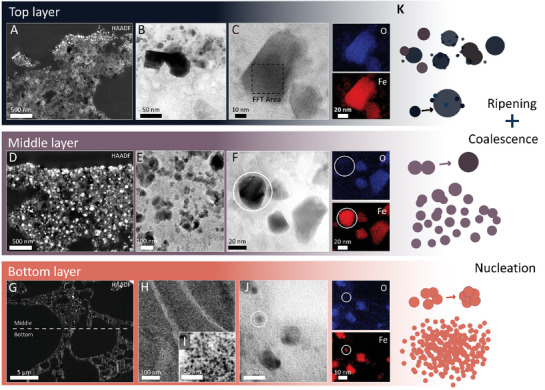
(S)TEM images (ultra‐thin sections) of the three layers, along with their microstructural features, corresponding iron phase, and associated energy‐dispersive X‐ray spectroscopy analysis (EDS). Specifically, Figure 5A,D,G provide a high‐angle annular dark‐field image (HAADF) overview of the top, middle, and bottom layers, respectively. The top layer is characterized by highly graphitized carbon (nano)ribbons, while magnetite (Fe_3_O_4_) particles B) are only found on the surface (C). EDS analysis C) and FFT (Figure S10, Supporting Information) were used to identify the iron phase. In the middle layer, iron particles are homogeneously dispersed D,E) in a graphitic matrix, while EDS F) and SAED (Figure S10, Supporting Information) helped to identify magnetite and γ‐iron phases. The bottom layer displays uniformly distributed magnetite particles H,I) characterized by small diameters ≈10 nm, identified as magnetite, and small amounts of oxygen‐free iron nanoparticles by EDS J) and corresponding SAED of (H) in Figure 6F. In the EDS images, O denotes oxygen while Fe denotes iron. K) shows a schematic representation of the individual particle movement within each individual layer.

**Figure 6:**
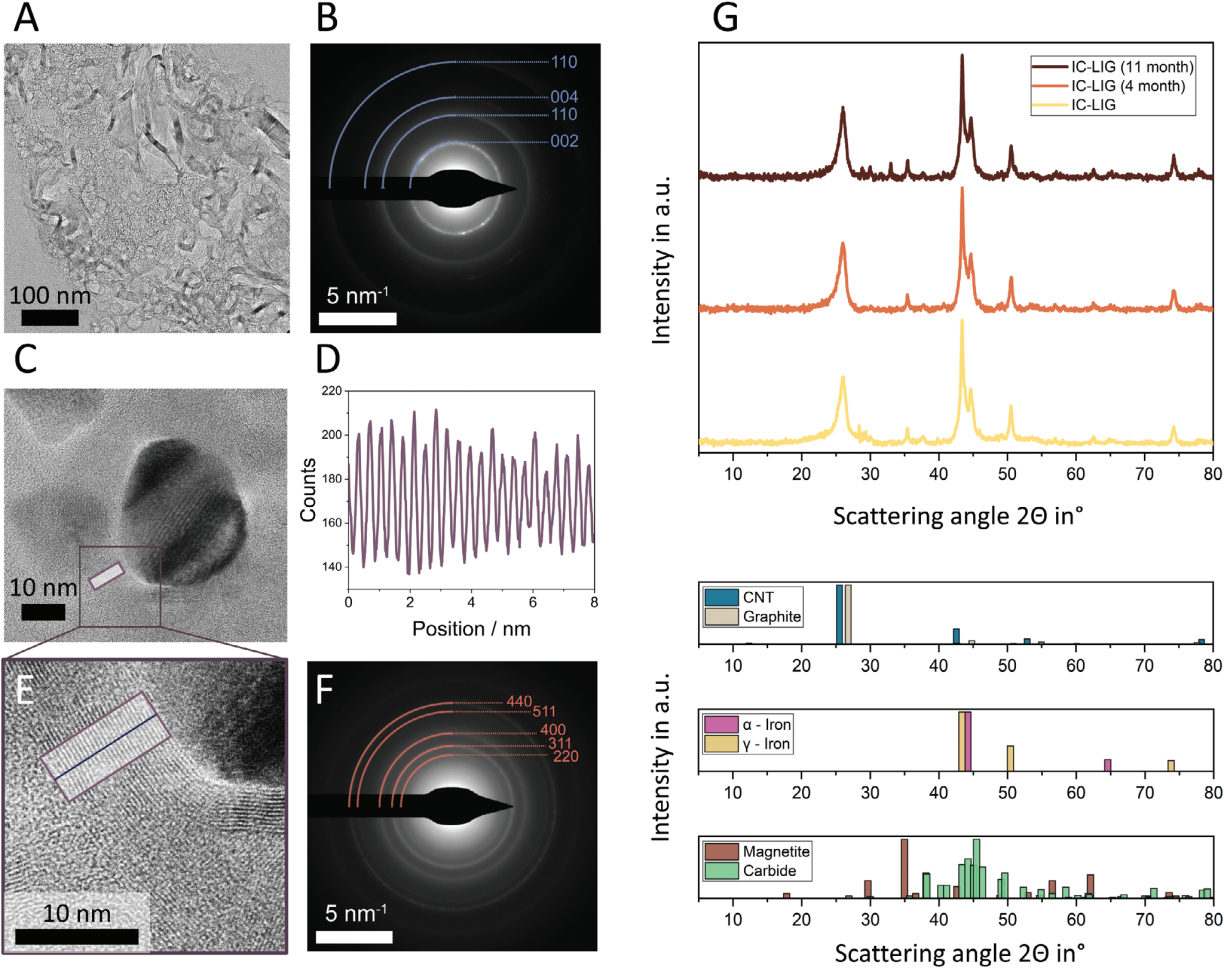
Analysis of high‐resolution TEM images (ultra‐thin sections) on the left and the crystallographic analysis together with reference diffractograms on the right. The highly graphitized top layer A) with the associated SAED together with the corresponding indexing of graphite B) reveals an interlayer spacing of 0.343 nm. An encapsulated γ‐iron particle with graphitic shell C) and its line plot D) derived from the intensity changes of the lattice fringes highlights an interlayer spacing of 0.358 nm (E, blue line), within the middle layer. The SAED F) corresponding to Figure 5H reveals that nanoparticles within the bottom layer are magnetite. Crystallographic analysis G) shows that the reference iron phases (bottom) conform with the recorded diffractograms (top), which highlights the stabilization of γ‐iron particles (distinct peak at 50.5°) over almost one year.

On the upper surface of the top layer, we observed irregularly distributed bright spots in the 3D nano‐CT model (Figure 2; Figure S3 and Movie S1, Supporting Information) as well as large metallic aggregates within the top layer of thin‐cut samples in the (S)TEM images (**Figure** **5**A). High‐resolution TEM images with EDS mapping (Figure 5C), as well as their associated rotational average of the Fourier transformed energy spectrum (Figure S10, Supporting Information), revealed that these irregularly shaped aggregates on top of a highly graphitized layer consist of magnetite particles. These large magnetite particles were surrounded by a significant fraction of much smaller magnetite particles, observed within high‐resolution TEM images as well as with atomic force microscope (AFM) analysis (Figure 5B; Figure S13, Supporting Information). Another distinctive feature of the top layer is a large amount of highly graphitized carbon nanoribbon structures (**Figure** **6**A; Figure S10E–G, Supporting Information). Figure 6A shows an overview of a high‐resolution TEM micrograph representing the carbon nanoribbons found in the top layer. The analysis of the corresponding selected area diffraction (Figure 6B) revealed an interplanar spacing of 0.343 nm, similar to highly graphitized carbon.^[^ ^26^ ^]^

It is important to note that the iron‐rich particles migrated outside the carbonaceous matrix, mainly toward the heat source and the upper surface of the top layer (Figure 5A). Hoekstra et al. investigated the pyrolysis of microcrystalline cellulose spheres (100–200 µm) loaded with iron(III) chloride.^[^ ^27^ ^]^ The authors observed the formation of large iron particles on the surface of the graphitized spheres due to the volatility of smaller iron nanoparticles, along with Ostwald ripening. Therefore, it is reasonable to assume that Ostwald ripening is the dominant phenomenon of nanoparticle sintering and movement in the top layer due to the highest energy input of the laser treatment.

### 2.2 Middle Layer

The aggregation of magnetite nanoparticles on the upper surface of the top layer is one of the main distinctive features compared to the middle layer underneath, where the iron‐rich particles are well dispersed in a highly graphitized matrix composed mainly of nanoribbons (Figure 5D,E). Here, some particles are even encapsulated in multilayer graphitic shells (referred to as “graphite onions” in the literature)^[^ ^28^ ^]^ (Figures 5F and 6C, Figure S10C, Supporting Information). The interplanar spacing of the graphitic shell was 0.358 nm, measured between single graphene sheets ((002) lattice fringes of ordered graphitic regions) (Figure 6C purple box, Figure 6D), similar to carbon lattice fringes of ordered graphitic regions derived from hard carbons.^[^ ^26^ ^]^ Interestingly, these encapsulated nanoparticles are mostly oxygen‐free, as identified by SAED and EDS maps (Figure 5F white circle, Figure S10 and S11, Supporting Information). Moreover, the X‐ray diffraction (XRD) analysis (Figure 6G) suggests the presence of γ‐iron due to the clear agreement of the recorded diffractograms with the reference peaks at 43.4°, 50.5°, and 74.2°. It is important to note that the stabilization of γ‐iron nanocrystals at room temperature has been reported. Here, γ‐iron as the core within a complex core‐shell structure was encapsulated by an amorphous iron carbide interface together with a few layers of graphene as the outermost shell.^[^ ^29^ ^]^ Considering the observed core‐shell structure together with the characteristic nano‐twinning^[^ ^30^, ^31^ ^]^ for γ‐iron nanoparticles observed by analysis of the fast Fourier transformation of an encapsulated oxygen‐free iron nanoparticle (Figure 5C; Figure S10C,D, Supporting Information) it seems reasonable to assume that we stabilized the γ‐iron particles in a graphitic shell. Moreover, no changes were observed between the diffractograms when collecting diffractograms directly after the IC‐LIG process and after 11 months of aging under atmospheric conditions (Figure 6G).

The temperature reached within the middle layer is a decisive factor for nanoparticle formation and migration. Aguiló‐Aguayo et al. (2013) found that the migration of iron from the carbon shell occurred at temperatures above 600 °C. The iron that had been released coalesced with other iron particles in close proximity, forming iron clusters to further reduce their surface energy.^[^ ^32^ ^]^ It is noteworthy that in (S)TEM images revealed a considerable amount of particle agglomeration and twin boundaries (Figure 5F, Figure S10C,D, Supporting Information), which suggests the coalescence of two nanoparticles.^[^ ^33^ ^]^ Consequently, in the middle layer, particle migration and coalescence are considered to be the primary mechanisms underlying the observed particle movement and growth. However, we assume that due to the highly dynamic situation during laser graphitization, both particle migration with coalescence and Ostwald ripening may occur simultaneously within the middle and top layers.

### 2.3 Bottom Layer

Following the structural changes within the 3D nano‐CT model toward the substrate, a decrease in intensity is observed at the transition from the middle to the bottom layer (Figure 5G; Figure S3 and S9, Supporting Information). The bottom layer is characterized by a much smoother structure with uniform, mostly closed, pores (Figure 2B, bottom layer top view images). We observed a significant quantity of dispersed iron particles (with sizes ranging from 3 to 7 nm) embedded in an amorphous carbon matrix (Figure 5H–J). In some instances, we observed small oxygen‐free nanoparticles (Figure 5J). However, the SAED of the bottom layer indicates that magnetite is the prevalent iron phase (Figure 6F). By high‐angle annular dark‐field imaging (HAADF) (Figure 5G white line), we also observed the boundary between the middle and bottom layers clearly defined by the sudden appearance of bright spots in the middle layer. The heat accumulated within the bottom layer is restricted by the unidirectional laser treatment, which limits the growth of magnetite nanoparticles in the bottom layer. We consider magnetite to nucleate along with the decomposition of the precursor ink already in the transition between the intumescence and carbonization zones. A nucleation phenomenon similar to the one we observed in our system (Figure 5J,K) was previously reported for a precursor system composed of gelatin and iron(II) acetate that has been graphitized using a conventional furnace.^[^ ^34^ ^]^

In light of the prospective applications in the domain of sustainable electronics, it is of significant interest to address the correlation between structural development and electrical conductivity. A detailed examination of the individual layers reveals a gradual increase in graphitization from the substrate toward the top layer, as evidenced by the increased carbon content in the EDS map (Figure 4; Figure S8, Supporting Information) and high‐resolution TEM images (Figure 6A). A high degree of graphitization, in conjunction with the percolation path, is directly related to the ability to conduct electricity. Consequently, it is reasonable to conclude that the top layer and the middle layer are primarily responsible for electron transport, given their high degree of graphitization. Additionally, both layers establish an interconnected, well‐defined percolation path, as evidenced by the supporting Raman measurements from the top view (Figure S7, Supporting Information). Here, the interconnected graphitized domains, which are characterized by I_D_ /I_G_ ratio of 0.15 to 0.85 (G‐Peak position: 1580 cm^−1^, D‐Peak position: 1348 cm^−1^) similar to that of graphitic carbons with an Intensity I_D_/I_G_ ratio of 0.1 to 0.8^[^ ^34^, ^35^, ^36^ ^]^ enabling low sheet resistance of 23.4 ± 1.6 Ω◻^−1^.

### 2.4 Graphitization Mechanisms of IC‐LIG

In our previous study, we showed that excluding iron from the ink resulted in inhomogeneous and amorphous carbon foams with sub‐optimal electrical properties (sheet resistance 60–70 Ω ◻^−1^).^[^ ^4^ ^]^ These findings underline the fundamental role of iron in the formation of a highly graphitized and electrically conductive layer during the laser‐induced graphitization process. Therefore, we assumed that the proposed mechanisms of catalytic graphitization based on transition metals for hydrothermal and pyrolytic treatments of organic precursors may also apply to our IC‐LIG treatment.^[^ ^26^, ^34^, ^37^, ^38^ ^]^

According to this interpretation, the iron‐tannic acid complex decomposes in the heat‐affected zone, promoting the formation of iron oxide (Fe_3_O_4_) nanoparticles. These nanoparticles are converted into iron carbide and eventually, at higher temperatures, into iron by carbothermal reduction. The catalytic graphitization of the amorphous carbonaceous matrix is driven by these iron‐rich particles.

For the catalytic graphitization to occur, the size^[^ ^39^ ^]^ and phase^[^ ^26^ ^]^ of the catalyst particles, as well as the prevailing temperature, and reaction time are important requirements. Thus, a direct comparison between iron‐catalyzed graphitization using conventional (furnace‐based) thermal treatments with laser‐induced processes seems challenging, given the differences in thermal input, elevated temperatures, and reaction times. Time, in particular, must be taken into account, which in the case of conventional thermal treatments can range from minutes to hours,^[^ ^37^ ^]^ while the reaction time, as defined by the presence of the laser beam at a spot, is in the millisecond range,^[^ ^21^ ^]^ as schematically illustrated in Figure 3D.

However, it is important to consider that the actual reaction of the catalytic graphitization occurs within several seconds when the required boundary conditions are reached, as observed by in situ measurements.^[^ ^40^, ^41^, ^42^ ^]^ Moreover, with the defocusing of the laser beam, we pass the same position several times with the laser, leading to the accumulation of heat (extension of the reaction time) necessary to graphitize the precursor (Figure 3F). Based on our structural analysis results, it is reasonable to conclude that the necessary catalyst conditions for initiating catalytic graphitization are present within the top and middle layers during laser graphitization.

Here, the top layer experiences the greatest amount of energy due to the heterogeneous energy input of the laser treatment. Therefore, the catalyst is expected to be mainly pure iron, as the iron carbide phase (partially) decomposes into metallic iron above 750 °C.^[^ ^26^ ^]^ We also observed a considerable amount of graphitic nanoribbons in the top layer (Figure 5A; Figure S10, Supporting Information). Surprisingly, the IC‐LIG process results in a high graphitization degree with an average interplanar spacing of 0.343 nm in the top layer, even higher than for conventional heat treatments reported (0.36 ± 0.01 nm in (002)).^[^ ^26^ ^]^ The formation of the nanoribbons is related to the fast movement of the catalytic iron nanoparticles during laser graphitization. Similar structures have been observed in conventional (thermal) metal‐catalyzed graphitization processes^[^ ^26^, ^37^ ^]^ in which an observed “liquid‐like” movement has been shown to leave traces of graphitic carbon nanotubes, as evidenced by in situ measurements.^[^ ^37^, ^43^ ^]^ It is worth noting that the catalyst particle is not in a liquid state. Recent in situ measurements have revealed that the catalytic graphitization process occurs via the rapid diffusion of a crystalline catalyst particle, which is in the solid state, through a solid amorphous carbon matrix.^[^ ^40^ ^]^

Moreover, in situ measurements reported that catalyst nanoparticles do not move back into the developed graphitized tubes, indicating that once graphitization is complete, graphitic carbon does not redissolve into the catalyst nanoparticles.^[^ ^37^, ^41^ ^]^ This may further explain the appearance of large iron nanoparticles (Ostwald ripening) and their tendency to aggregate in the upper surface of the top layer observed within the IC‐LIG electrodes. Upon cooling, the iron nanoparticles can react with the oxidative atmosphere, forming larger magnetite particles. This is supported by thermogravimetric analysis of the iron‐tannic acid ink conducted in an inert nitrogen atmosphere (N₂) and an oxygen‐rich atmosphere, as well as by the corresponding X‐ray diffractograms of the residuals (Figure S12, Supporting Information).

The laser‐graphitization condition in the middle layer is different, which directly influences the structure. Here, we observed the formation of nanoribbons together with encapsulated iron nanoparticles, by means of graphitic shells. The ribbon‐like structures are likely a result of the catalytic nanoparticle movements, similar as previously described for the top layer. However, the temperature in the middle layer is anticipated to be lower than that in the top layer, therefore, we expect iron carbide to be the catalyst. The theory of carbon precipitation during catalytic graphitization of supersaturated iron carbide particles has already been described.^[^ ^41^ ^]^ According to this theory, the surrounding amorphous carbon matrix is dissolved and precipitates as more ordered graphitic structures due to the associated reduction in free energy, while the movement of the iron carbide (nano) particles can further lead to traces of hollow graphitic tubes and channels.^[^ ^26^, ^41^ ^]^ Here,size effects and the structure of the amorphous carbon matrix as a precursor are assumed to significantly lower the required temperature to allow a catalytic graphitization, while after cooling, the reported iron phase is α‐iron in the bcc structure.^[^ ^26^ ^]^

However, instead of α‐iron, we stabilized γ‐iron in a core‐shell arrangement, as evidenced by the X‐ray spectroscopy analysis and the (S)TEM observations (Figures 5F and 6C,E, Figure S10, Supporting Information). Here, we expect that saturated iron carbide precipitates carbon, forming a graphitic shell. The encapsulated iron core remains in the γ‐iron phase (as the intermediate phase)^[^ ^26^ ^]^ instead of transforming into ferrite (α‐iron in bcc structure), which would require a much slower cooling rate (10 °C min^−1^).^[^ ^26^ ^]^ Here, the heat dissipation in dependency of the structure needs to be considered as well. Comparable to the heat dissipation through the sample thickness, the horizontal heat transport depends on the thermal coefficient of the materials present. Within the carbonization zone, amorphous carbon acts as a thermal insulator^[^ ^24^ ^]^ and prevents the induction of excessive heat in the plane (Figure 3E), while the fully graphitized IC‐LIG electrode in the lag zone of the laser consists of highly graphitized carbon nanostructures, which is expected to rapidly dissipate the induced heat and act as a heat sink.^[^ ^44^ ^]^ In this context, ultrafast pulsed lasers, which are distinguished by a relatively small focal area and a high repetition rate, offer the potential for more precise control of the laser energy profile and, consequently, the induced heat. These lasers have already been demonstrated to facilitate the direct graphitization of wood or cellulose‐based materials with minimal damage.^[^ ^3^, ^25^ ^]^ Consequently, the precise control of the catalytic iron phase and nanoparticle size could be envisioned with such laser systems.

### 2.5 Comparison of IC‐LIG with Catalyst‐Free Laser Graphitization

The use of a laser, with its distinctive high energy density at the focal point (Figure 3F), presents a significant challenge to the substrate material or precursor employed, especially for temperature‐sensitive materials. Usually, a successful laser‐induced graphitization of bio‐based materials can be achieved only by employing special pre‐treatments, laser parameters, and dedicated laser setups to prevent ablation or thermal damage to the material.^[^ ^3^, ^15^, ^45^ ^]^ The use of iron‐tannic acid ink represents a notable advantage in this regard. The ink treatment is limited to the surface, as it penetrates only the upper layers (≈20 µm) of the substrate. Thus, the substrate becomes a carbon precursor itself, as observed by the nano‐CT image series (Figure S4, Supporting Information). Here, the coated wood fibers are transformed into the IC‐LIG electrode, becoming a carbonaceous foam while facilitating a uniform transition from the substrate toward the IC‐LIG electrode, determining the highly porous structure.

A direct structural comparison of IC‐LIG materials with those obtained by catalyst‐free laser graphitization is difficult since the structural variations in LIG are as rich as their processes, precursors, and substrates used. However, thanks to catalytic graphitization, IC‐LIG exhibits a high specific surface area of ≈74.5 m^2^·g⁻¹ as evaluated by BET (N₂ adsorption) (sheet resistance of 23.4 ± 1.6 Ω◻^−1^), in comparison to ≈3.19 m^2^g^−1^ and 7.26 m^2^g^−1^ for LIG derived from wood and wood/MnO^[^ ^46^ ^]^ and 4.6 m^2^g^−1^ (sheet resistance 46 ± 3 Ω◻^−1^for LIG derived from cork.^[^ ^47^ ^]^ This is especially interesting considering future applications since both, LIG from wood/MnO and cork have been used for energy storage devices.

## 3 Conclusion

An in‐depth multiscale analysis was conducted to elucidate the catalytic graphitization mechanisms of iron‐catalyzed laser‐induced graphitization. The entire IC‐LIG electrode was preserved by embedding samples in a low‐viscosity resin that allowed us to cut ultrathin cross‐section (≈100 nm) with an ultra‐microtome. Consequently, the IC‐LIG process was virtually frozen in time within the thin cuts and their associated block face samples. With complementary characterization techniques including nano‐CT analysis, Raman spectroscopy, EDS and (S)TEM, AFM, and XRD analysis, a comprehensive 3D reconstruction was obtained incorporating the nanoscale findings and placing them in a mesoscopic perspective. We identified a layered arrangement of the IC‐LIG electrode across its thickness, consisting of three distinct layers, namely the top, middle, and bottom layers. While the top and middle layers consist of a highly graphitized carbon matrix, with an averaged interlayer spacing of ≈0.343 nm, the bottom layer consists of amorphous carbon. This heterogeneous graphitization degree is related to the unidirectional energy derived from the laser treatment. Magnetite was identified as the prevailing iron phase within all layers; however, our results also indicate a significant amount of γ‐iron within the middle layer due to the rapid cooling associated with the laser treatment. It is interesting to note that the stabilization of γ‐iron at room temperature was achieved through a simple laser treatment in an ambient atmosphere. Additionally, a special focus on the growth and migration behavior of the iron catalyst nanoparticles allowed us to identify Oswald ripening in the top layer, migration with coalescence in the middle layer, and nucleation within the bottom layer. Eventually, we compare our laser‐induced graphitization process with conventional transition metal catalysis‐based processes to illustrate the fundamental differences and mechanistic similarities between these two methods. By contrasting catalyst‐free laser graphitization approaches with IC‐LIG we were able to highlight the unique properties and structural features of IC‐LIG electrode.

The study offers a fundamental understanding of the structural evolution and graphitization mechanism for metal‐catalyzed laser‐induced graphitization, as well as a versatile preparation technique applicable to the production of multilayer electrode materials. The insights gained are of value to research endeavors in various fields, including electrodes for energy storage devices, catalysts, sensors, and beyond. Further research is needed to identify the mechanisms by which the different iron phases within each layer can be controlled and the structure modified, as well as to study the influence of these modifications on application‐relevant properties such as porosity, electrical conductivity, and catalytic activity.

## 4 Experimental Section

### Materials

Tannic acid (source: Chinese natural gall nuts), ammonium iron (III) citrate (technical grade), and glycerol (99+%) were purchased from Sigma‐Aldrich. All chemicals were used as received. Deionized water (DI) was thoroughly used unless otherwise stated. As substrate material, acid‐free wood pulp board (Gebr. Bühler AG Zürich, CH) produced from mechanical pulp of pine, spruce, and birch was used. The wood pulp board had a double‐sided smooth surface and a grammage of 750 g m^−^^2^, while its thickness was ≈1.5 mm.

### Preparation of the Iron‐Tannic Acid Ink

The composition of the iron‐tannic ink presented in the first study^[^ ^4^ ^]^ was simplified by avoiding gum Arabic and using ammonium iron(III) citrate instead of iron(III) citrate (Figure 1). Since ammonium iron(III) citrate is water soluble already at room temperature, the energy required for ink processing was reduced, well in line with green chemistry principles. Thus, Tannic acid (33 g) was first dissolved by adding it in small portions under stirring (≈500 rpm) to 77 g of deionized water. With continued stirring, 8 g of glycerol, and eventually 10.2 g of ammonium iron(III) citrate were added sequentially in small portions to ensure their complete dissolution. The resulting iron‐tannic acid ink stored was at room temperature until use.

### Ink Deposition on Substrates

The iron‐tannic acid ink was applied on wood pulp board samples using a commercial paintbrush (GraduateXL, flat, Daler Rowney, UK). Three layers of ink were applied to obtain a homogenous coating, resulting in a 100 µm thick layer, measured with a micrometer (Mitutoyo). The applied ink amount was measured with a laboratory scale (AE 163, Mettler, CH) resulting in 170 g m^−2^. The samples were then left to dry for at least 12 h at 20 °C and 65% RH before use. A figure of the coated surface is attached to the supporting information (Figure S14 and Table S1, Supporting Information).

### Laser Treatment

Samples were treated with a commercial 10.6 µm CO_2_ laser engraver (Speedy 400, Trotec). The laser has a maximum power of 80 W and a maximum scan rate of 4.3 m s^−1^. The used parameter for the laser treatment depended on the substrate. Thus, the applied power was 7.5% with a scan rate of ≈200 mm s^−1^, an image density of 1000 pulses inch^−1^, and a defocus up to 5 mm (resulting beam diameter of ≈450 µm).

### Characterization Techniques

Analytical (scanning) transmission electron microscopy ((S)TEM) was performed on an FEI Talos F200X operated at 200 kV acceleration voltage. The specimens were dried at 103 °C for 12 h and embedded in Spurr low viscosity embedding resin (Sigma Aldrich). Ultrathin sections (≈100 nm) were prepared on an ultramicrotome (Ultracut E, Reichert‐Jung) equipped with a diamond knife (Diatome) and flattened using chloroform vapor. The ultrathin sections were deposited on copper grids with a ≈5 nm continuous carbon supporting film. Elemental content distribution mapping was carried out by energy‐dispersive X‐ray spectroscopy (EDS) STEM spectrum imaging with a windowless Super‐X EDS system. The EDS spectra were acquired up to 20 keV with a spectral resolution of 10 eV per channel and an acquisition time of 300 s to 1200 s. Selected‐area electron diffraction (SAED) patterns were acquired with a 40 µm SAD aperture corresponding to a diameter of ≈800 nm in the object plane. The corresponding reference cards for analysis of the SAED patterns were PDF 04‐015‐2407 for graphite and PDF 00‐019‐0629 for magnetite.

High‐resolution block‐faces images of carbon‐coated samples (2 nm) were taken with a scanning electron microscope (SEM, SU5000, Hitachi Ltd., Japan) driven by an accelerating voltage of 2 kV, while the local concentrations of iron and oxygen were mapped with the energy‐dispersive X‐ray detectors (Oxford Ultim Max 100 EDS detectors mounted at 90° to each other, Oxford Instruments, UK) driven by a 10 kV acceleration voltage. The EDS analysis was conducted using the software AZtec version 6.1 SP2 (Oxford Instruments), which was employed to display the individual phases in an image overlay (map mode). To achieve optimal differentiation between the phases, the weighting of oxygen, carbon, and iron was divided equally, and the brightness was adjusted accordingly. The individual raw images of the specific phases are provided in the Supporting Information (Figure S8, Supporting Information).

A PANalytical X'Pert PRO MPD diffractometer using monochromated Cu Kα1 radiation (40 kV, 45 mA) in Bragg–Brentano geometry was used. Samples for X‐ray diffraction (XRD) were prepared by scratching off the LIG from samples of the size 20 × 20 mm^2^, further ground using an agate pestle and mortar, and mounted on a low background Si sample holder. By optimizing the pulse height distribution limit (PHD, lower limit set to 60%, upper limit to 80%), the fluorescent background was suppressed. The diffractograms were collected from 5° to 80° with a total acquisition time of 5 h and a step size of 0.0334°. A background was fitted and subtracted, and peaks were indexed, using HighScore Plus. The corresponding reference cards from the PDF4 database^[^ ^48^ ^]^ are 00‐041‐1487 for graphite,^[^ ^49^ ^]^ 00‐058‐1638 for carbon nano tubes,^[^ ^50^ ^]^ 00‐006‐0696 for α‐iron, 04‐003‐1443 for γ‐iron, 00‐019‐0629 for magnetite, and 00‐35‐0772 for iron carbide.

For the nano‐CT acquisition, cubic samples with the size of 1 × 2 × 1.5 mm^3^ were cut with a CO_2_ laser engraver (Speedy 400, Trotec) and mounted on a needle tip with 2K epoxy (Z‐Poxy, ZAP, USA). They have been scanned focusing through a zoom scan on the interface surface area using an EasyTom XL Ultra 230 160 micro/ nano CT scanner (RX Solutions, Chavanod, FR). The scanner operated at 60 kV and 165 µA. The samples were scanned over a 360° rotation with a step size of 0.1°, frame average of 3, and exposure time of 2 s. The nominal resolution was set at 250 nm voxel size. CT images were reconstructed using X‐Act (RX Solutions, Chavanod FR) software.

Reconstructed 2D CT slices were then analyzed using AVIZO software (Thermo Fisher Scientific, USA). This software was used to generate 3D models, MIP projections, and porosity calculations. In detail, the porosity percentages of each slice were evaluated using the corresponding layer's rectangular bounding box, which encloses the individual layer in its entire volume. A global threshold was applied for the segmentation of the porous structure into its distinct regions based on pixel intensity. Thus, the box represents the total volume V_Total_, from which the volume of the individual layer V_Layer_ is subtracted to obtain the porosity Φ according to Equation (1):

(1) $$\Phi =\frac{V_{Total}-V_{Layer}}{V_{Total}}\times 100\%$$

Raman spectroscopy for chemical mapping of the cross‐section was performed with a confocal Raman microscope (Renishaw InVia) using a 785 nm laser, an objective (Zeiss, 20x), and a 1200 l mm^−1^ grating (maximal measurement range 1100 cm^−1^). The integral exposure time was 1 s for 50 accumulations covering a spectral range of ≈1000–2100 cm^−1^ with ≈2.4 mW laser power (5% of 48 mW) for single‐point measurements. As mapping parameters, an integration time of 1 s for 3 accumulations with 2.4 mW laser power (5% of 48 mW) covering a spectral range of ≈1000–2100 cm^−1^, and a step width of 750 nm was used in the Map image acquisition mode over an area of ≈350 × 150 µm^2^ resulting in 94432 measurement points. After data acquisition, a baseline correction and cosmic ray removal filter were applied, while acquired spectrums were normalized (0,1) for subsequent chemical image analysis using the Wire 3.7 software (Renishaw UK). For chemical imaging, data were exported into CytoSpec (v. 2.00.01), a commercially available MatLab‐based software. The built‐in vertex component analysis tool (https://www.cytospec.com/multivar.php#ImageVCA) has been used as an unsupervised method to rapidly unmix hyperspectral data. By covering a spectral range of ≈1000–2100 cm^−1^, a set of distinct spectral features of each component (endmember) was measured, thereby enabling a clear distinction between them. Here a total of five endmembers, representing the total set of all components in the sample, were used, while its signal‐to‐noise ratio was set to 0.005. Single endmembers were represented as abundance plots (monochromatic images), wherein a high color intensity corresponds to a high contribution of the individual endmember. All endmember abundance plots were plotted, converted to RGB format, and overlaid, resulting in a colored map of the analyzed area (Figure S6B, Supporting Information). The corresponding line plots of the Raman data (Figure S6A, Supporting Information) were representative Raman spectra for the individual endmembers, respective identified material.

Raman spectroscopy for a single point as well as for a map for top‐view measurements, a 532 nm laser, an objective (Zeiss, 20×), and an 1800 l mm^−1^ grating was used. The integral exposure time was 3 s for 25 accumulations covering a spectral range of 1220–2790 cm^−1^ with 5 mW laser power (5% of 100 mW) for single point measurements. As mapping parameters, an integration time of 3 s (single spectrum acquisition) with 5 mW laser power (5% of 100 mW) and a step width of 1 µm over an area of ≈200 × 100 µm^2^ were used in the Map image acquisition mode. After data acquisition, a baseline correction and cosmic ray removal filter were applied using the Wire 3.7 software (Renishaw UK). For chemical imaging, data were exported into CytoSpec (v. 2.00.01). The integrated intensity ratio of the G‐peak band (1500–1650 cm^−1^) D‐peak band (1280–1400 cm^−1^) was used to obtain a color‐scaled representation of the Raman map (200 × 200 µm^2^). Intensity ratios of single point measurements and ROIs were evaluated using the software OriginPro 2019 (version 9.6.0.172, OriginLab Corporation, US).

Thermogravimetric analysis (Q50, TA instruments, New Castle, USA) with a platinum sample pan was used to compare the effects of an inert nitrogen atmosphere (N_2_) and an oxygen‐rich atmosphere (synthetic air; N_2_ + 20% ± 2% O_2_) during thermal treatment (up to 1000 °C, 10 °C min^−1^) of the untreated iron‐tannic acid ink.

Quantitative imaging (QI) measurements were conducted on ultramicrotome‐polished block face samples using an atomic force microscope (AFM) NanoWizard 4 instrument (JPK Instruments AG – Bruker Nano GmbH, Germany) equipped with a polygon‐based pyramid silicon cantilever (CONTR‐10, Nano World, Switzerland). The samples were affixed to glass slides with the cross‐sectional cut facing upward, and the measurements were conducted in a 20 °C and 65% relative humidity environment. The cantilever's spring constant was calculated to be 0.27 N m^−1^ using the thermal noise method. Additionally, its sensitivity, measured at 55.8 nm V^−1^, was determined through a contact‐based method on a glass slide. The measurements were conducted at a rate of 100 ms, a setpoint voltage of 0.1 V. A scan size of 40 µm x 40 µm for overview mapping and 1 µm x 1 µm for detailed map images with a resolution of 256 × 256 pixels were applied. Data processing was performed using the JPK image processing software, developed by JPK Instruments AG – Bruker Nano GmbH, Germany.

The specific surface area (SSA) of the samples was determined through a multipoint Brunauer‐Emmett‐Teller (BET) analysis of nitrogen gas sorption isotherms, which were performed at 77 K by using a high vacuum adsorption analyzer (autosorb iQ, Quantachrome, US). Prior to the measurements, the samples were degassed at 100 °C under vacuum for at least 24 h.

Sheet resistivity measurements were conducted using a four‐point probe (Signatone 302, spring‐loaded (0.6N) round‐headed gold contacts) equipped with a source measure unit (2450, Keithley Instruments, US).

In general, data was further evaluated and illustrated using the software OriginPro 2019 (version 9.6.0.172, OriginLab Corporation, US) and Adobe Illustrator (version 28.4.1, Adobe Inc., US). The Scientific color map Lipari^[^ ^51^ ^]^ was used to prevent visual distortion of the data and exclusion of readers with colour‐vision deficiencies.^[^ ^52^ ^]^

## Conflict of Interest

The authors declare no conflict of interest.

## Supporting information



Supporting Information

Supplemental Movie 1

## Data Availability

The data that support the findings of this study are available from the corresponding author upon reasonable request.
