# Supplementary material for: Iron‐Catalyzed Laser‐Induced Graphitization – Multiscale Analysis of the Structural Evolution and Underlying Mechanism
Source: Small. 2024 Sep 16;20(49):2405558. doi: 10.1002/smll.202405558 (PMC11618722; doi:10.1002/smll.202405558)
Supplement: Supplementary file 1 — Supporting Information [file SMLL-20-2405558-s002.docx]

Supporting Information

**Iron‐Catalyzed Laser‐Induced Graphitization – Multiscale Analysis of the Structural Evolution and Underlying Mechanism**

*Christopher H. Dreimol*, Ronny Kürsteiner, Maximilian Ritter, Annapaola Parrilli, Jesper Edberg, Jonas Garemark, Sandro Stucki, Wenqing Yan, Susanna Tinello, Guido Panzarasa*, Ingo Burgert**

*Corresponding authors**

C. H. Dreimol, R. Kürsteiner, M. Ritter, J. Garemark, S. Stucki, W. Yan, G. Panzarasa, I. Burgert

Wood Materials Science, Institute for Building Materials, ETH Zürich, 8093 Zürich, Switzerland

E-mail: [cdreimol@ethz.ch](mailto:cdreimol@ethz.ch); [guidop@ethz.ch](mailto:guidop@ethz.ch); [iburgert@ethz.ch](mailto:iburgert@ethz.ch)

C. H. Dreimol, M. Ritter, S. Stucki, I. Burgert

Cellulose & Wood Materials Laboratory, Empa – Swiss Federal Laboratories for Materials Science and Technology, 8600 Dübendorf, Switzerland

A. Parrilli

Center for X-ray Analytics, Empa – Swiss Federal Laboratories for Materials Science and Technology, 8600 Dübendorf, Switzerland

J. Edberg

RISE Research Institutes of Sweden, Digital Systems, Smart Hardware, Bio- and Organic Electronics, Södra Grytsgatan 4, 60233, Norrköping, Sweden

S. Tinello

Laboratory for Multifunctional Materials, Department of Materials, ETH Zürich, 8093 Zürich, Switzerland


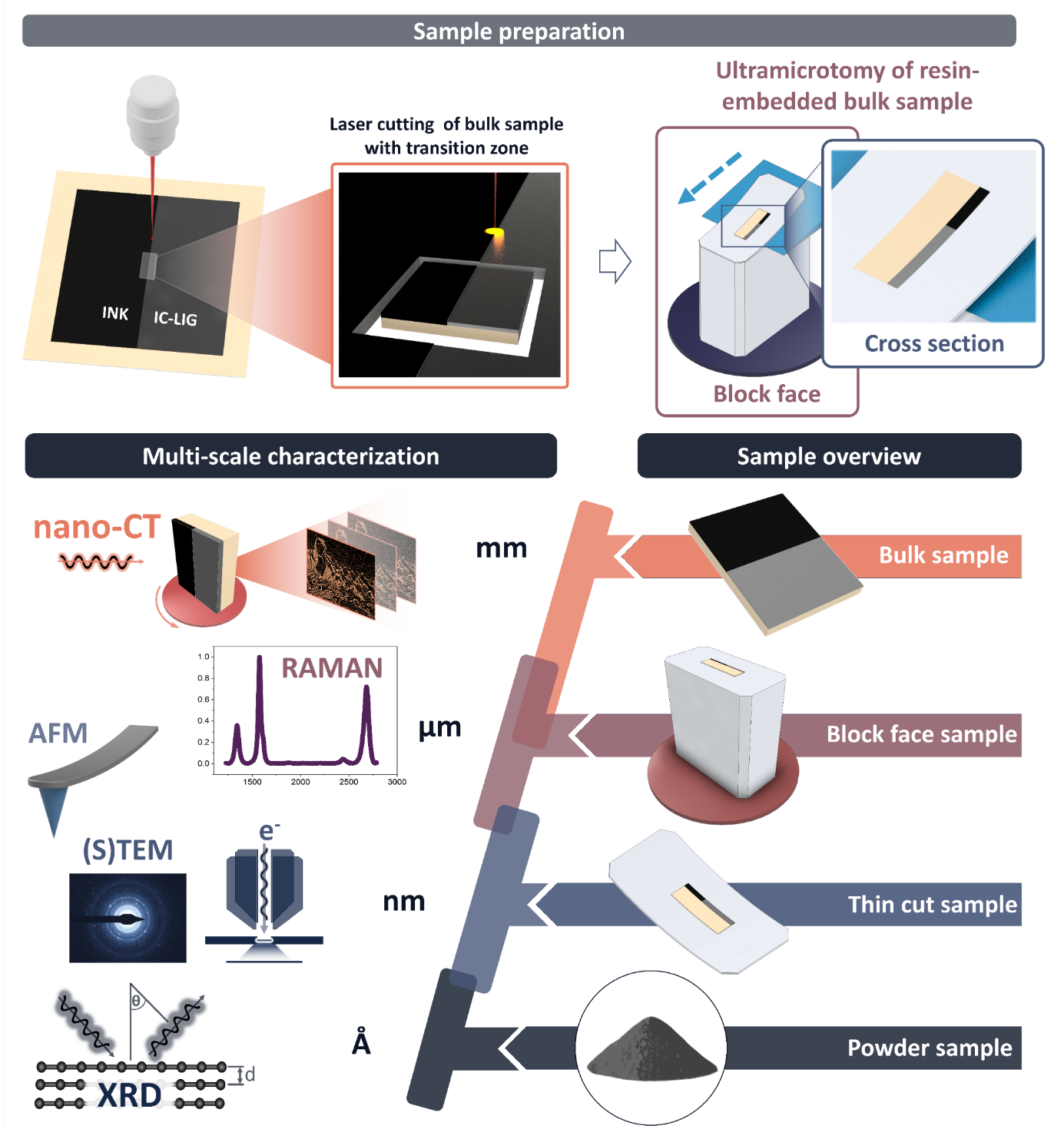


**Figure S 1** Schematic overview of the sample preparation and characterization methods. For mesoscale analysis using nano-CT, bulk samples from the transition zone, as well as fully graphitized samples, were laser cut. For nanoscale analysis, the laser-cut samples were resin-embedded and prepared using an ultra-microtome. Block face samples were used for Raman microscopy and SEM imaging, while thin cuts were used for (S)TEM analysis. X-ray diffractometry was performed on scratched-off powder samples.


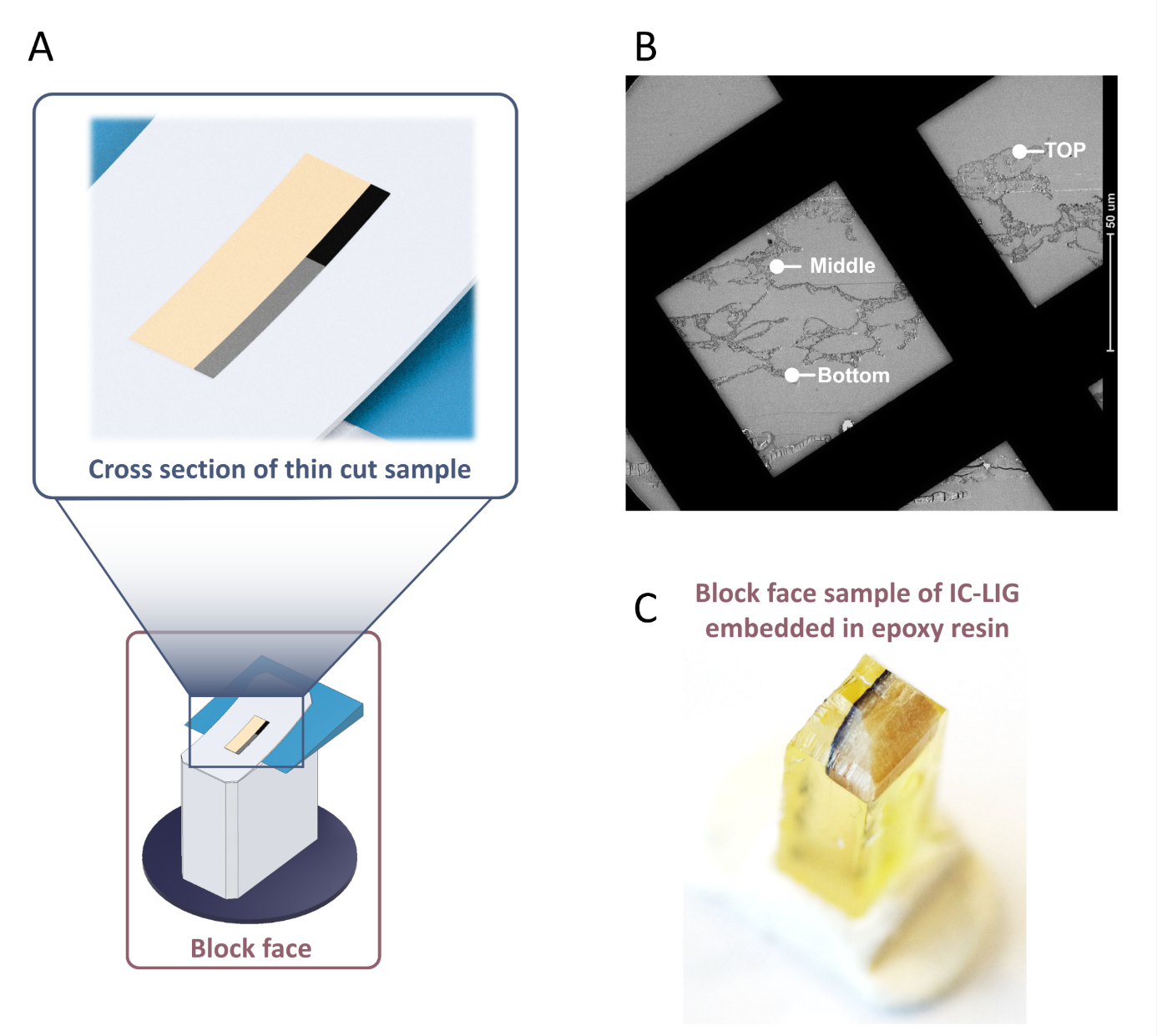


**Figure S 2** Schematic representation of the block face sample and thin cut (A). Thin cut sample on a TEM grid provides an overview of a sample showing all three layers (white points) (B). Block face sample embedded in epoxy resin shows the IC-LIG electrode (black layer) on the wood pulp board substrate (C).





**Figure S 3** Single image slices from Nano-CT analysis from top towards the substrate of the top view of the transition zone (A) and the fully graphitized area (B).


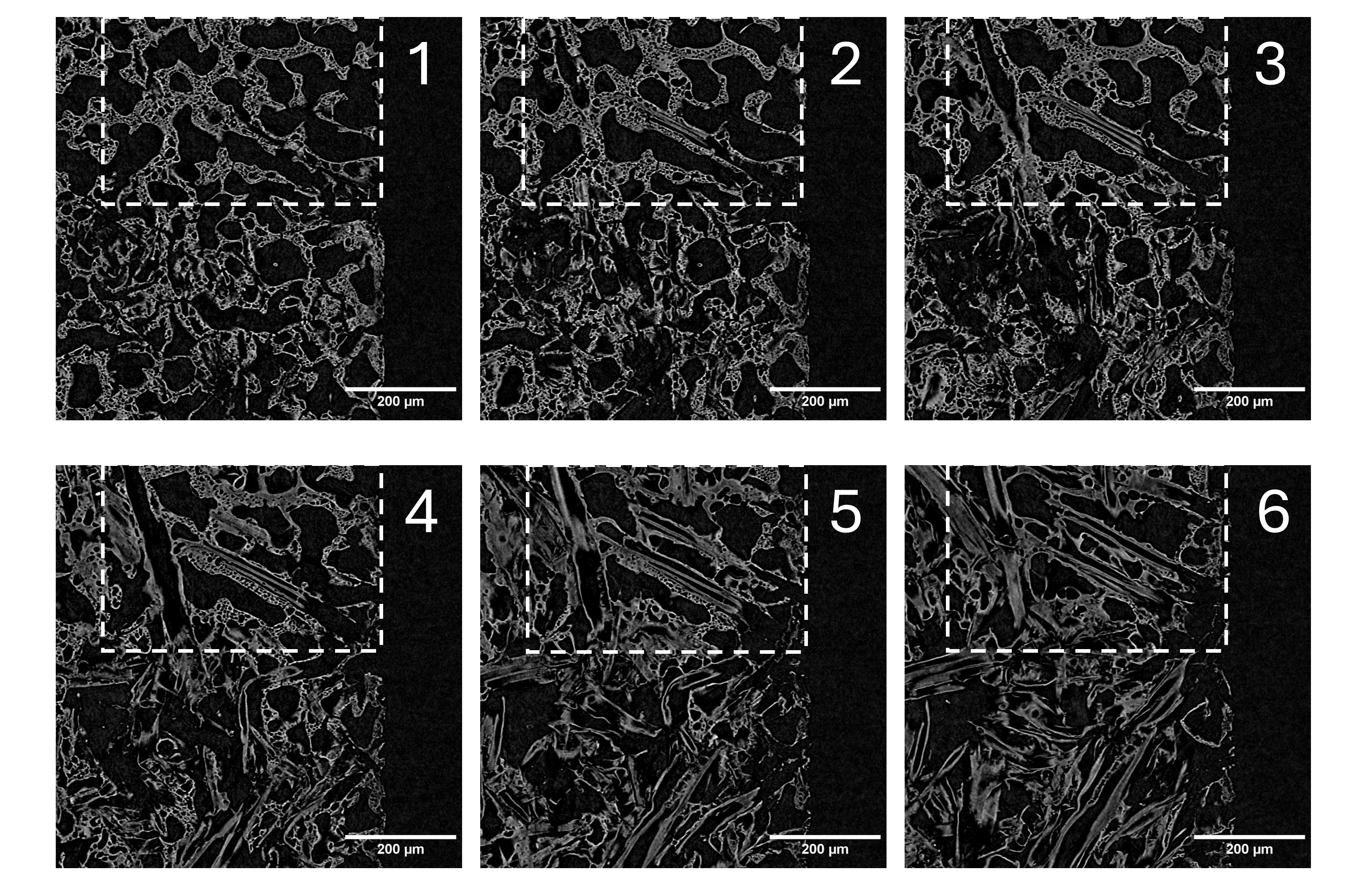


**Figure S 4** Nano-CT images of the amorphous carbon layer (1) in successive image sequences towards the substrate (6) wood pulp board. Some fibers themselves become a porous carbon structure (box with dotted lines), indicating that they serve as a direct precursor during IC-LIG.


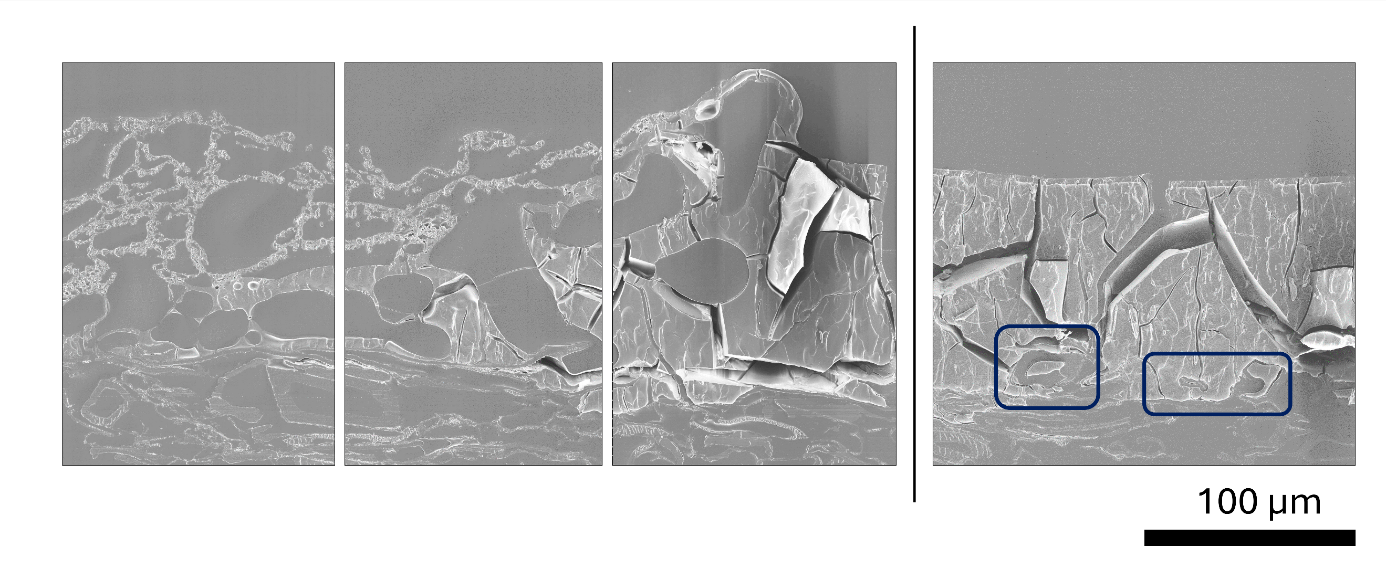


**Figure S 5** SEM images of the cross-section of the block face samples from the transition zone (left) and the ink precursor (right) are presented. The ink-encapsulated wood fibers (blue squares on the right image) within the applied ink layer facilitate the adhesion of the ink to the substrate and serve as a precursor for the subsequent laser graphitization.


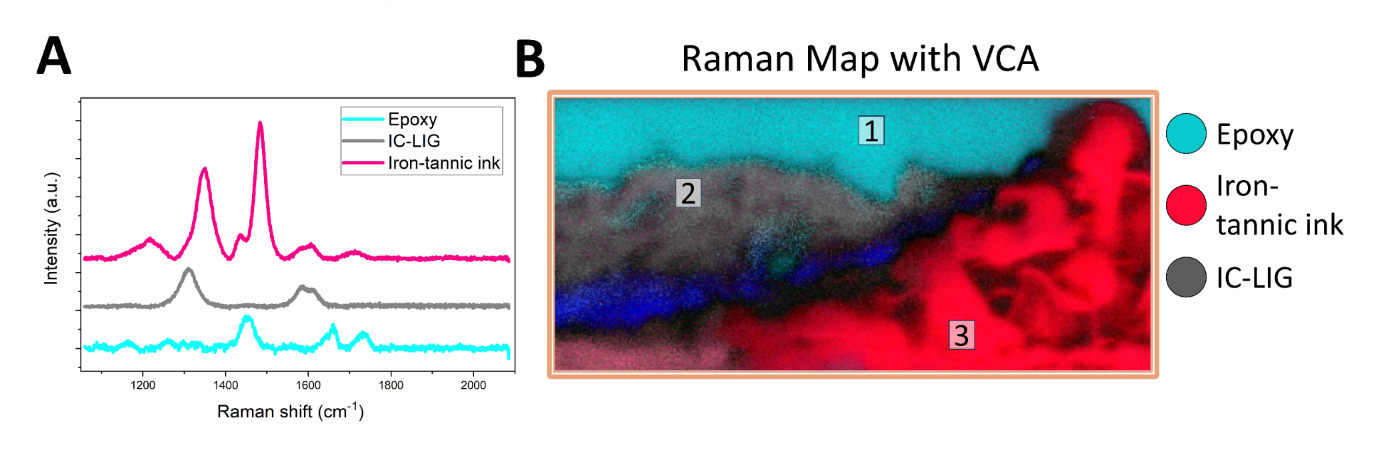


**Figure S 6** Confocal Raman measurements, which are represented by the spectra of the regions of interest (ROI) in (A) of the corresponding Raman map (B) from the SEM cross-section of the block face sample (**Figure 4A**). Vertex Component Analysis (VCA) was used to unmix the hyperspectral Raman data and discriminate between individual endmembers, thereby identifying the respective materials. By covering a spectral range of approximately 1000-2100 cm^-1^ and a step size of 750 nm, it was possible to obtain distinguishable spectra for each measurement point. Consequently, a color map (B) of the analyzed area shows an overlay of the colored endmember abundance plots, highlighting the individual materials identified in the transition zone. These include epoxy (petrol), IC-LIG (grey) and the iron tannic acid ink (red).


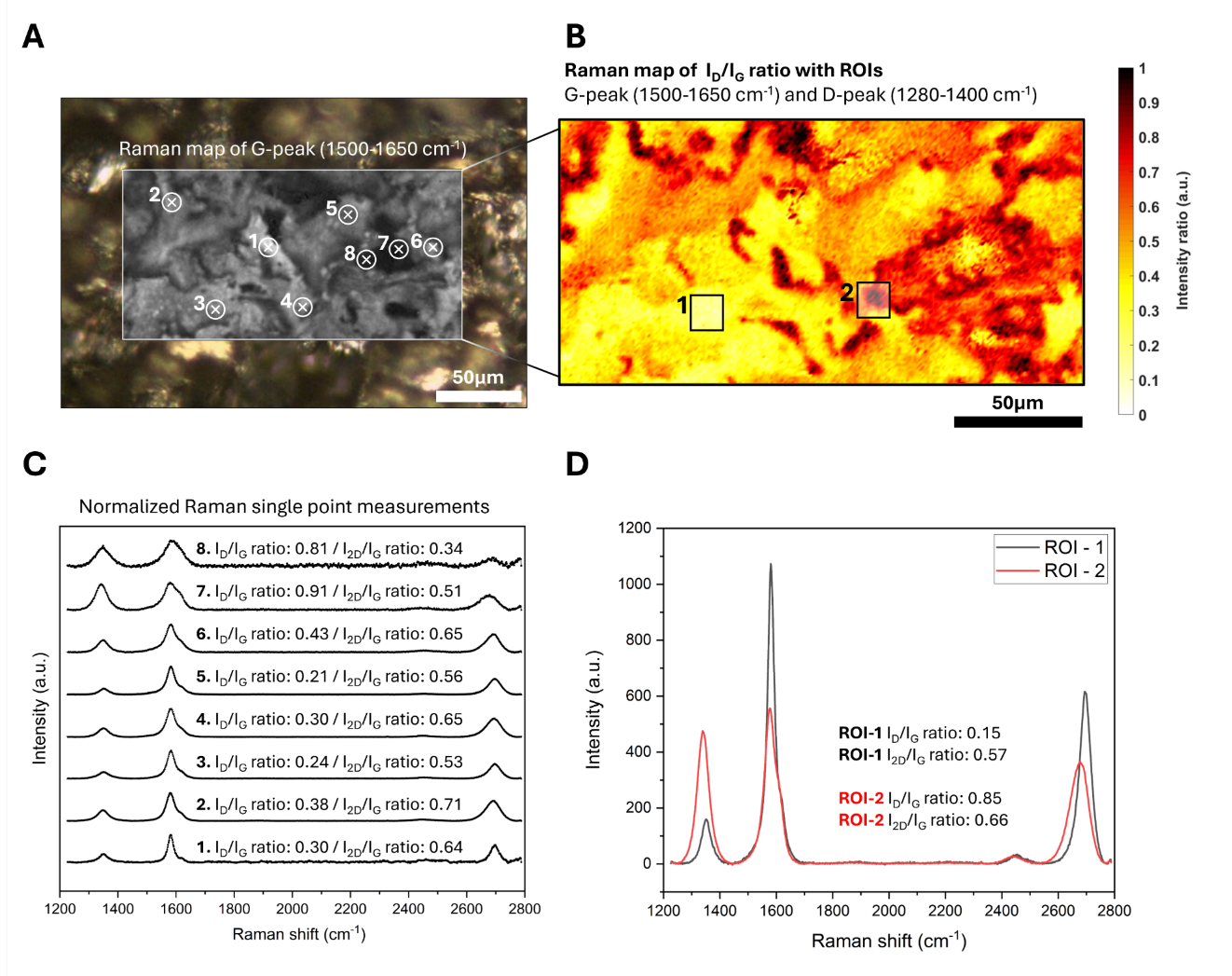


**Figure S 7** Raman map from the top view of fully graphitized IC-LIG showing the G*-*peak in (A) with the corresponding Raman map of the intensity ratio I_D_/I_G_ in (B). Single point measurements (C) with the corresponding spectra obtained from 1220 - 2790 cm^-1^ with the corresponding intensity ratio analysis of I_D_/I_G_ and I_2D_/I_G_ highlighting the areas with high degree of graphitization (1-6) and lower degree of graphitization (7,8) analogous to the region of interest (ROI) analysis (D) of the intensity ratio map I_D_/I_G_ in (B) showing with ROI-1 highly graphitized IC-LIG similar to that of graphite with an Intensity I_D_/I_G_ ratio of 0.1 to 0.8^[1]^ ^[2]^, and with ROI-2 lower graphitization more similar to that of amorphous carbon ^[3]^ ^[4]^ ^[5]^.


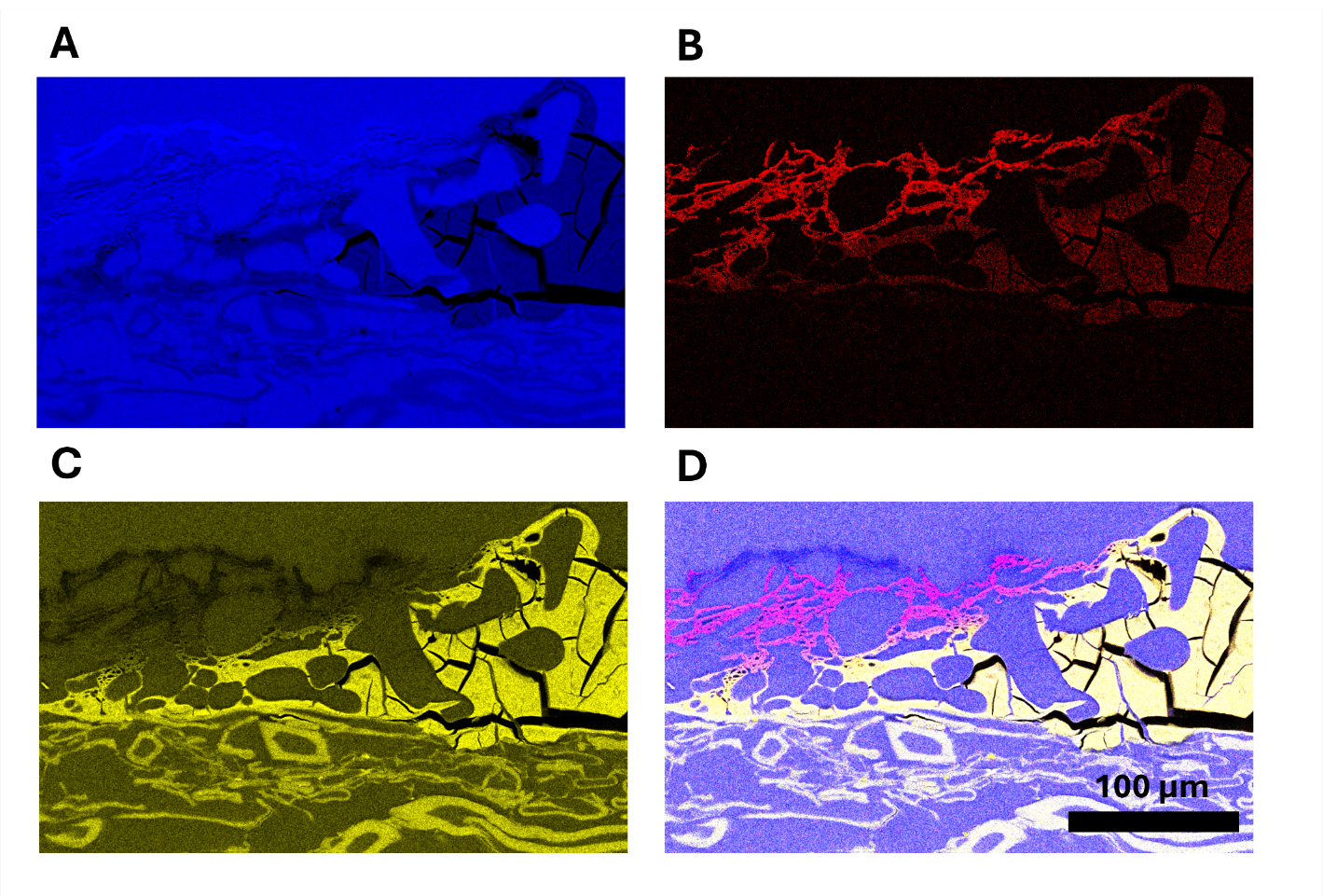


**Figure S 8** EDS maps of the transition zone with increased brightness to emphasize the distribution of the individual elements including carbon (A, blue), iron (B, red), and oxygen (C, yellow) with the corresponding image overlay.


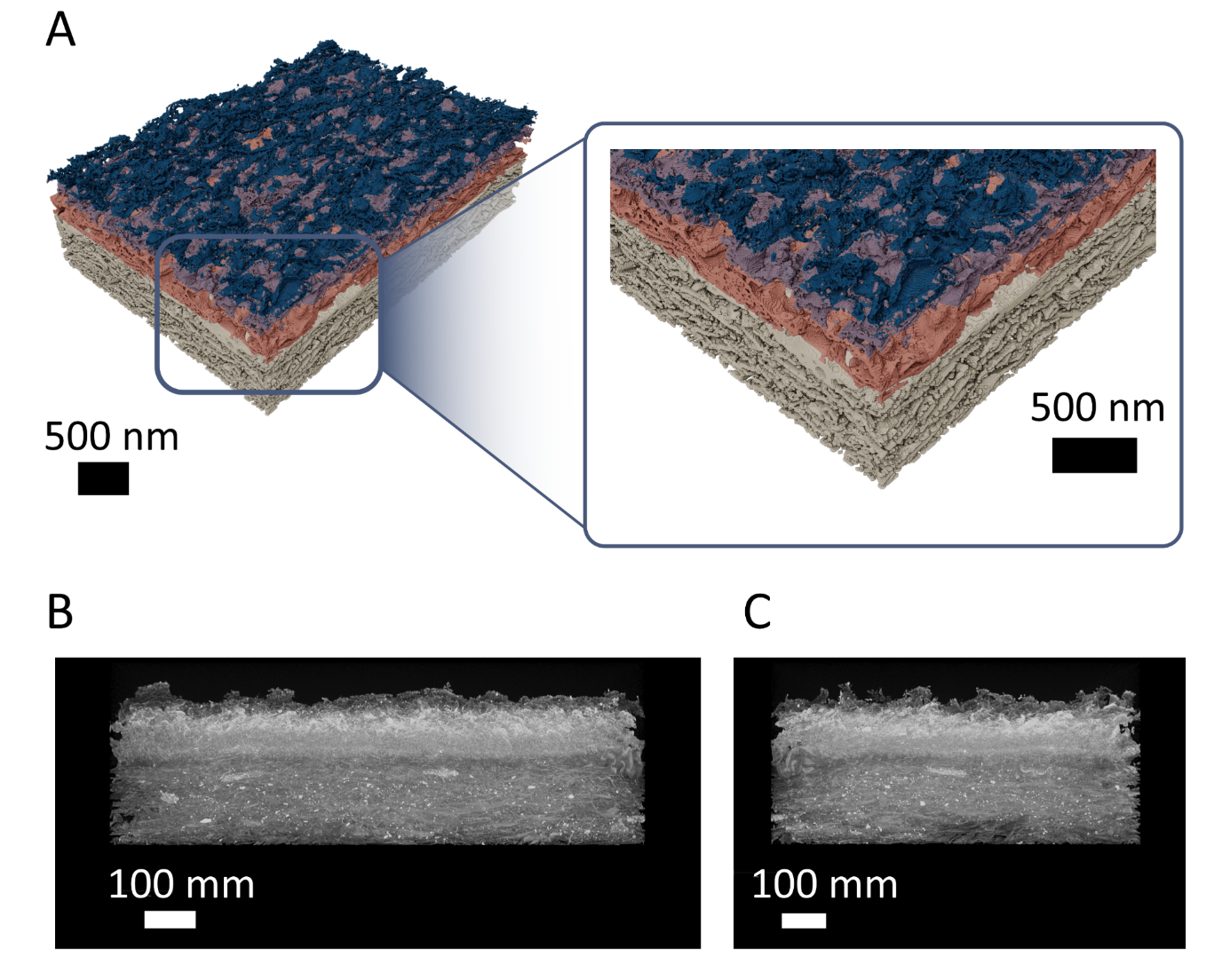


**Figure S 9** Separation of the individual layers of the IC-LIG electrode across its thickness (A). Inset shows the merging of the layers of individual layers along its interfaces. Maximum intensity projections of two perspective, long (B) and short (C) edge of the analyzed sample.


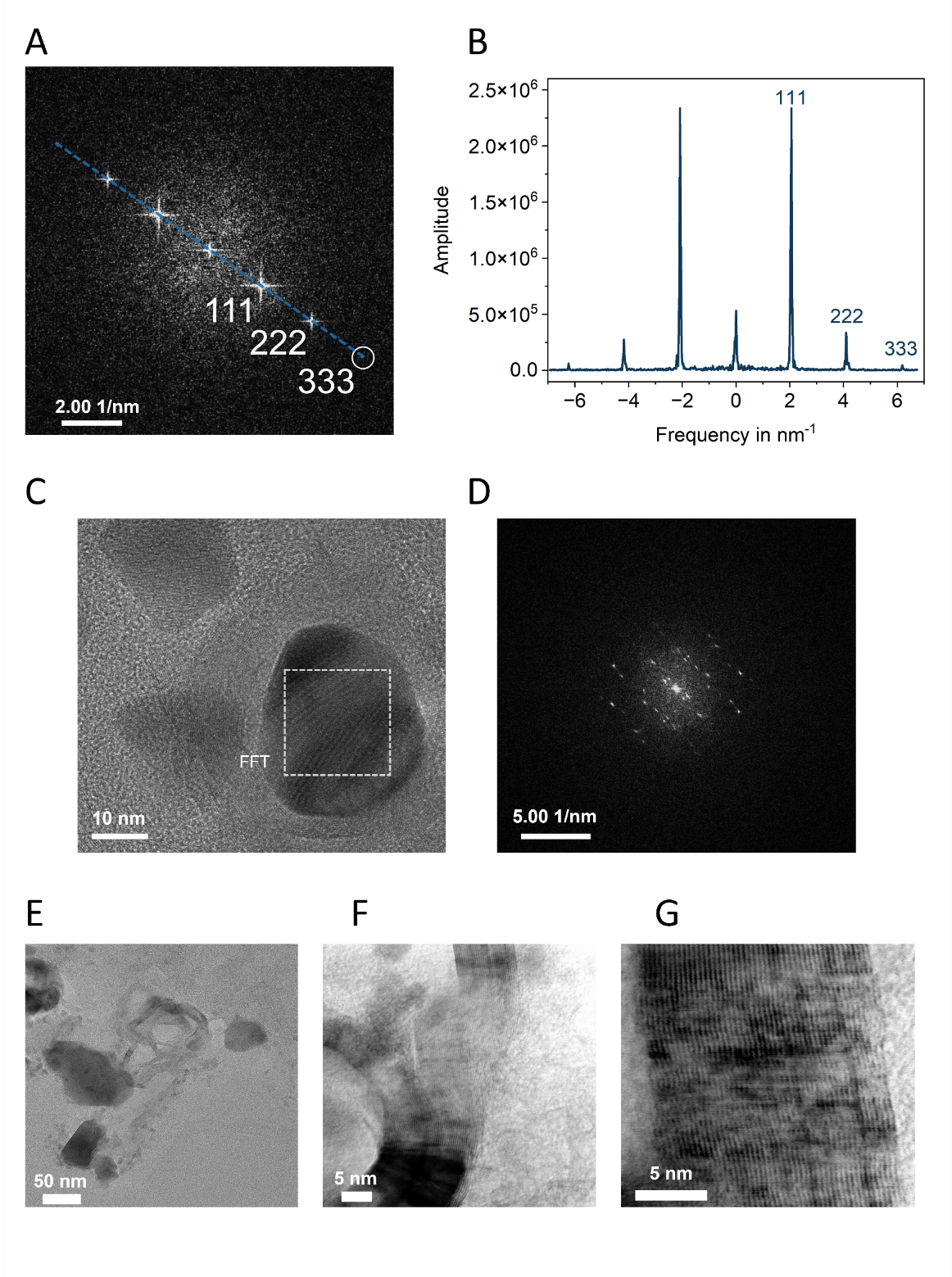
**Figure S 10** (A) shows the fast Fourier transform (FFT) of the high-resolution TEM image (**Figure 5C, FFT area**) with the indexed reflections of magnetite. (B) shows the line profile of (A) through the dashed blue line. In (C) the used FFT area for the FFT (D) highlights the nano-twinning of an γ-iron particle. In E-G, representative images for the highly graphitized top surface are provided.


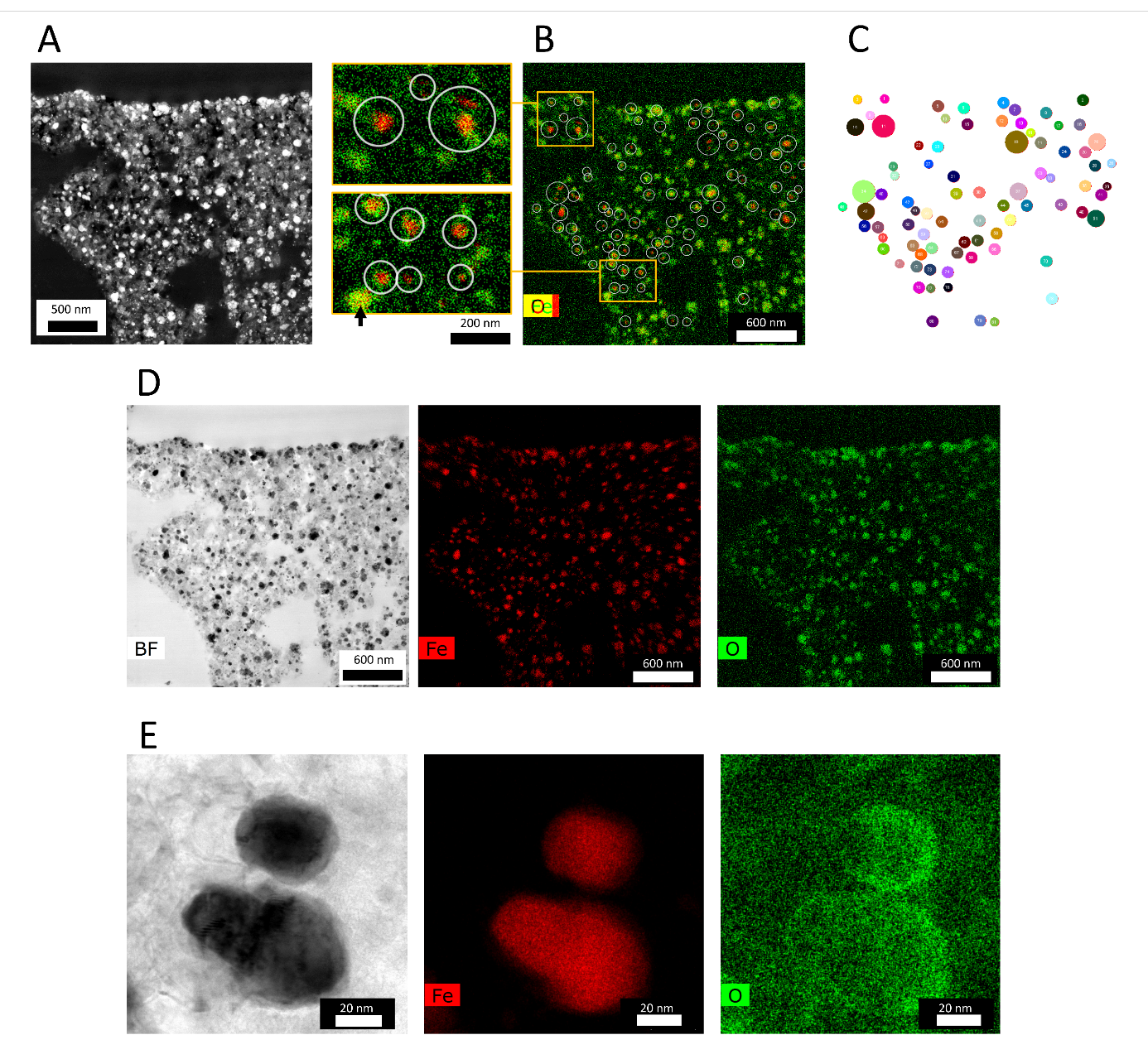


**Figure S 11** Overview image of the middle layer with the HAADF (A) and bright field (BF) image (D) with the corresponding EDS maps for iron (Fe) and oxygen (O). The overlay of the EDS maps (bottom iron, top oxygen) (B) with the magnified inset serves to highlight the oxygen-free iron phases, marked with white circles (total 81 positions). In comparison, the black arrow indicates an oxygen-rich iron nanoparticle. The coalescence of two individual particles can be seen from the bright field STEM image (E) with its associated EDS maps.


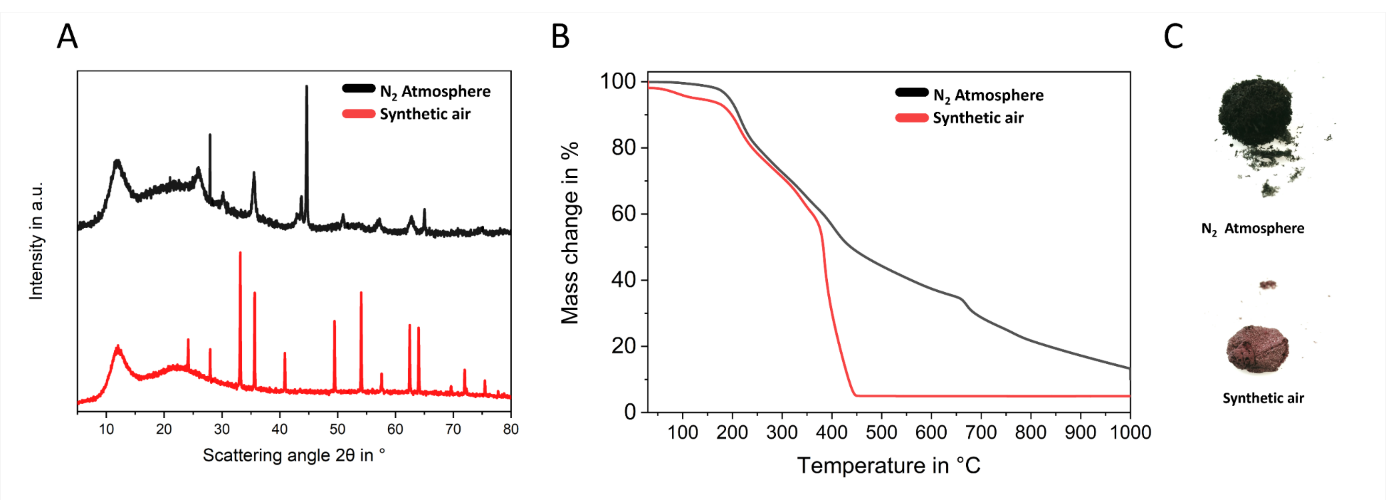


**Figure S 12** Crystallographic analysis (A) of the samples heat treated in the thermogravimetric analysis (B) in nitrogen (black) and synthetic air (red) atmosphere. Residual samples in (C).

Volatile gases produced during the decomposition of the iron-tannic acid ink may affect the local reaction atmosphere during laser-graphitization as well. It is well known that the overall properties of the resulting carbonaceous material in LIG depend on the used atmosphere.^[6]^ Therefore, we used thermogravimetric analysis to compare the effects of an inert nitrogen atmosphere (N_2_) and an oxygen-rich atmosphere. Thermal treatment under an inert nitrogen atmosphere resulted in the formation of a carbon-rich, highly porous composite (~12% of residual mass percent at 1000 °C), whereas under an oxygen-rich atmosphere a rust-red, nugget-like residue was formed (about 5% residual mass percent at 1000 °C) (**Figure S12**). X-ray diffraction confirmed the presence of carbon together with magnetite for inert nitrogen atmosphere (N_2_) and iron oxide (hematite) for an oxygen-rich atmosphere. This indicates that the iron particles on the upper surface of the top layer are undergoing oxidation in the surrounding atmosphere.


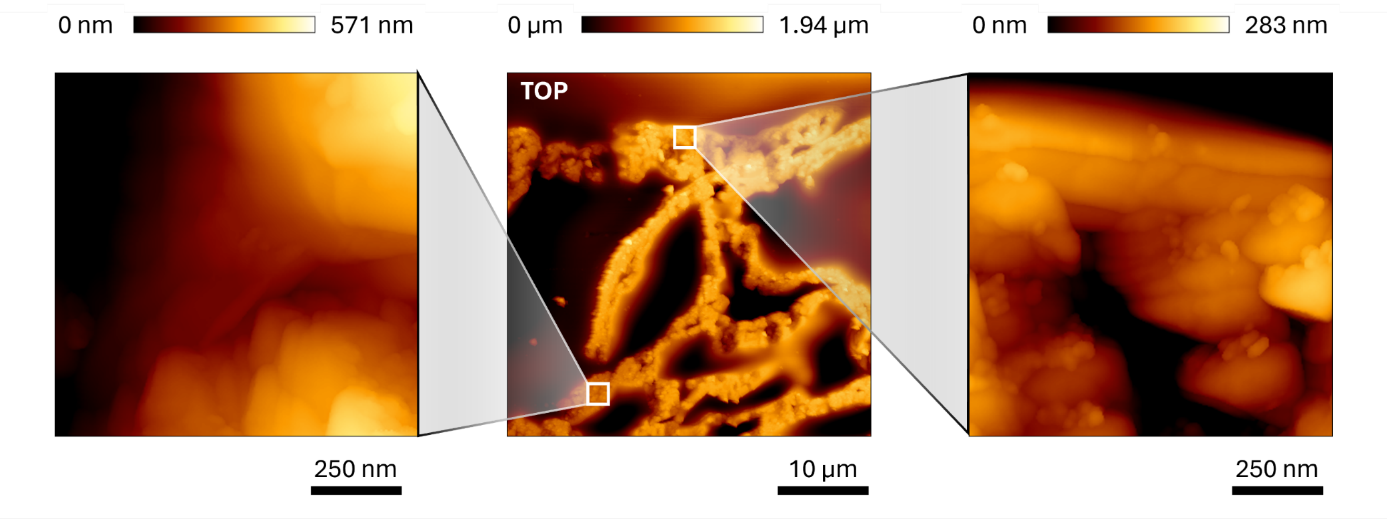


**Figure S 13** AFM height map of an ultramicrotome-polished cross-sectional block face sample of the transition from the top to the middle layer is presented, accompanied by detailed insets. The inset of the middle layer (left) displays a relatively smooth surface, while the inset of the top layer (right) illustrates the presence of distinct small particles surrounding larger ones.


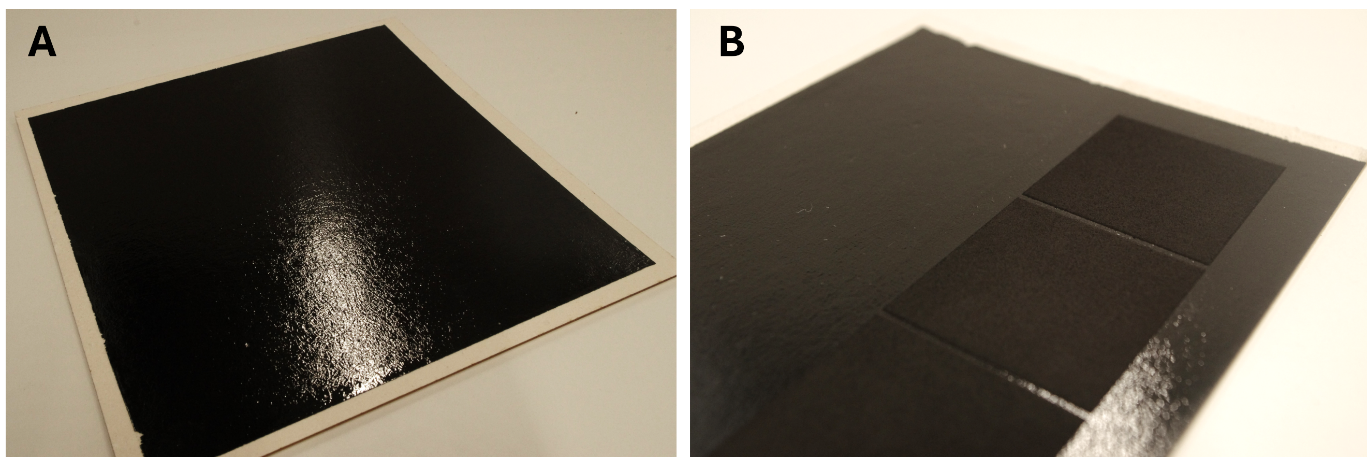


**Figure S 14** Ink-coated wood pulp board (20 cm x 20 cm) in (A) results in a 100 µm thick layer and approximately 170 gm² of applied ink. In (B), the homogeneous coating with laser-graphitized squares (40 x 40mm^2^) is highlighted, resulting in an electrically conductive IC-LIG electrode.

**Table S 1** Thickness measurements of the wood pulp board with and without an ink-coating with the resulting ink layer thickness and the results of electrical measurements.

**
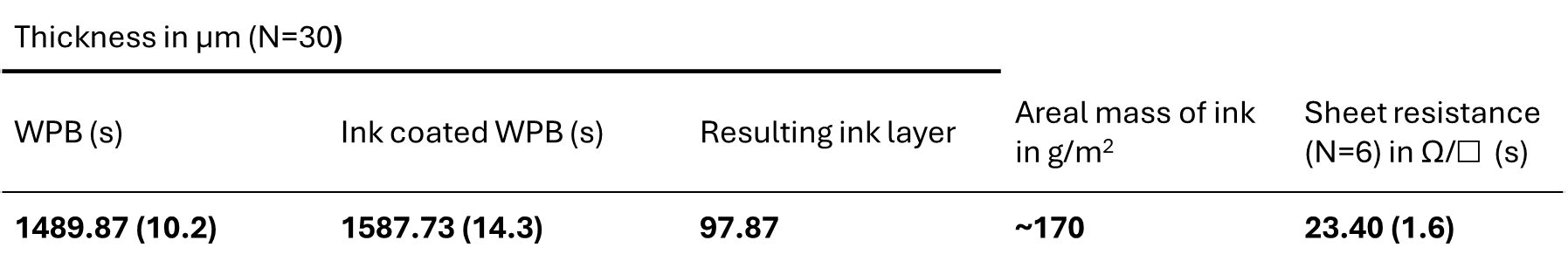
**

**References**

[1] D. B. Schuepfer, F. Badaczewski, J. M. Guerra-Castro, D. M. Hofmann, C. Heiliger, B. Smarsly, P. J. Klar, Carbon 2020, 161, 359.

[2] E. B. Barros, H. Son, G. G. Samsonidze, A. G. Souza Filho, J. Mendes Filho, G. Dresselhaus, M. S. Dresselhaus, Physical Review B 2007, 76.

[3] D. B. Schüpfer, F. Badaczewski, J. Peilstöcker, J. M. Guerra-Castro, H. Shim, S. Firoozabadi, A. Beyer, K. Volz, V. Presser, C. Heiliger, B. Smarsly, P. J. Klar, Carbon 2021, 172, 214.

[4] A. C. Ferrari, J. Robertson, Physical Review B 2000, 61.

[5] J. Schwan, S. Ulrich, V. Batori, H. Ehrhardt, S. R. P. Silva, Journal of Applied Physics 1996, 80, 440.

[6] M. Devi, H. Wang, S. Moon, S. Sharma, V. Strauss, Adv Mater 2023, 35, e2211054.
